# Correction: PSG9 Stimulates Increase in FoxP3^+^ Regulatory T-Cells through the TGF-β1 Pathway

**DOI:** 10.1371/journal.pone.0175636

**Published:** 2017-04-06

**Authors:** The PLOS ONE Staff

## Notice of Republication

This article was republished on February 10, 2017, to correct figure errors that were introduced during the composition process. The publisher apologizes for the errors. Please download this article again to view the correct version. The originally published, uncorrected article and the republished, corrected article are provided here for reference.

## Supporting information

S1 File Originally published, uncorrected article. (PDF)

S2 File Republished, corrected article. (PDF)
