# Supplementary material for: PSG9 Stimulates Increase in FoxP3+ Regulatory T-Cells through the TGF-β1 Pathway
Source: PLoS One. 2016 Jul 7;11(7):e0158050. doi: 10.1371/journal.pone.0158050 (PMC4936685; doi:10.1371/journal.pone.0158050)
Supplement: S2 File — (PPTX) [file pone.0158050.s002.pptx]

## Slide 1
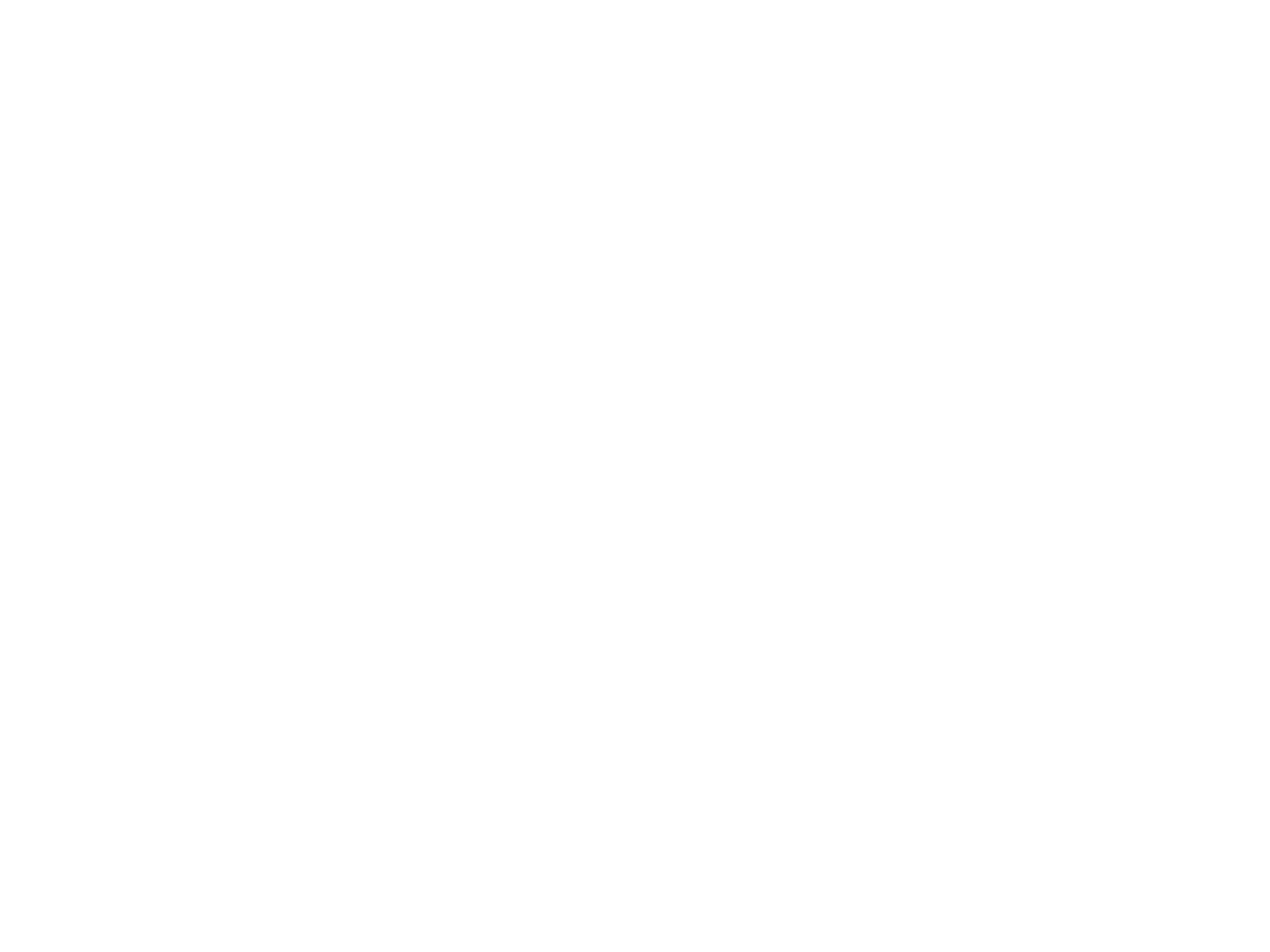

#

## Slide 2
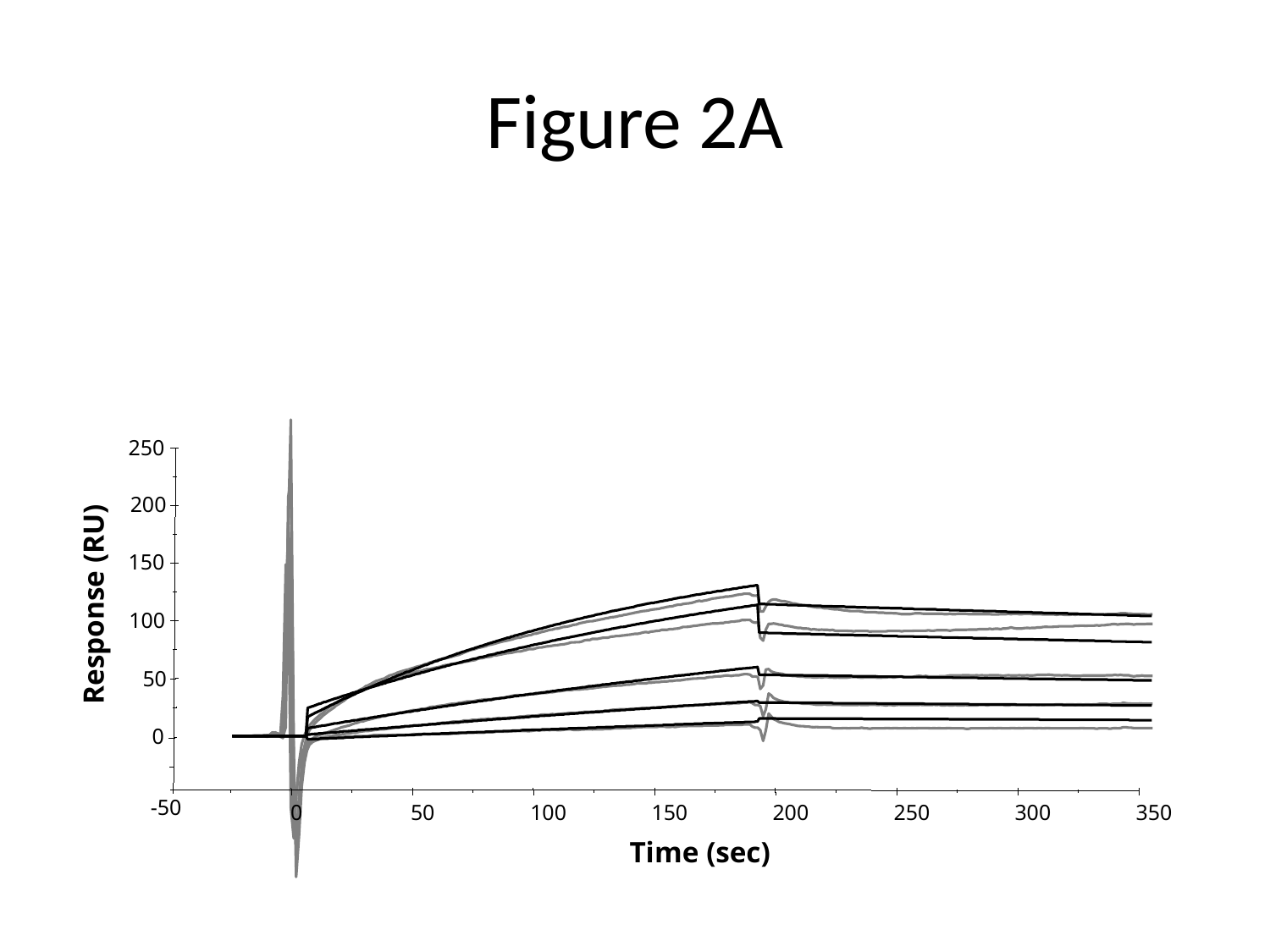

# Figure 2A
250
200
150
Response (RU)
100
50
0
-50
0
50
100
150
200
250
300
350
Time (sec)

## Slide 3
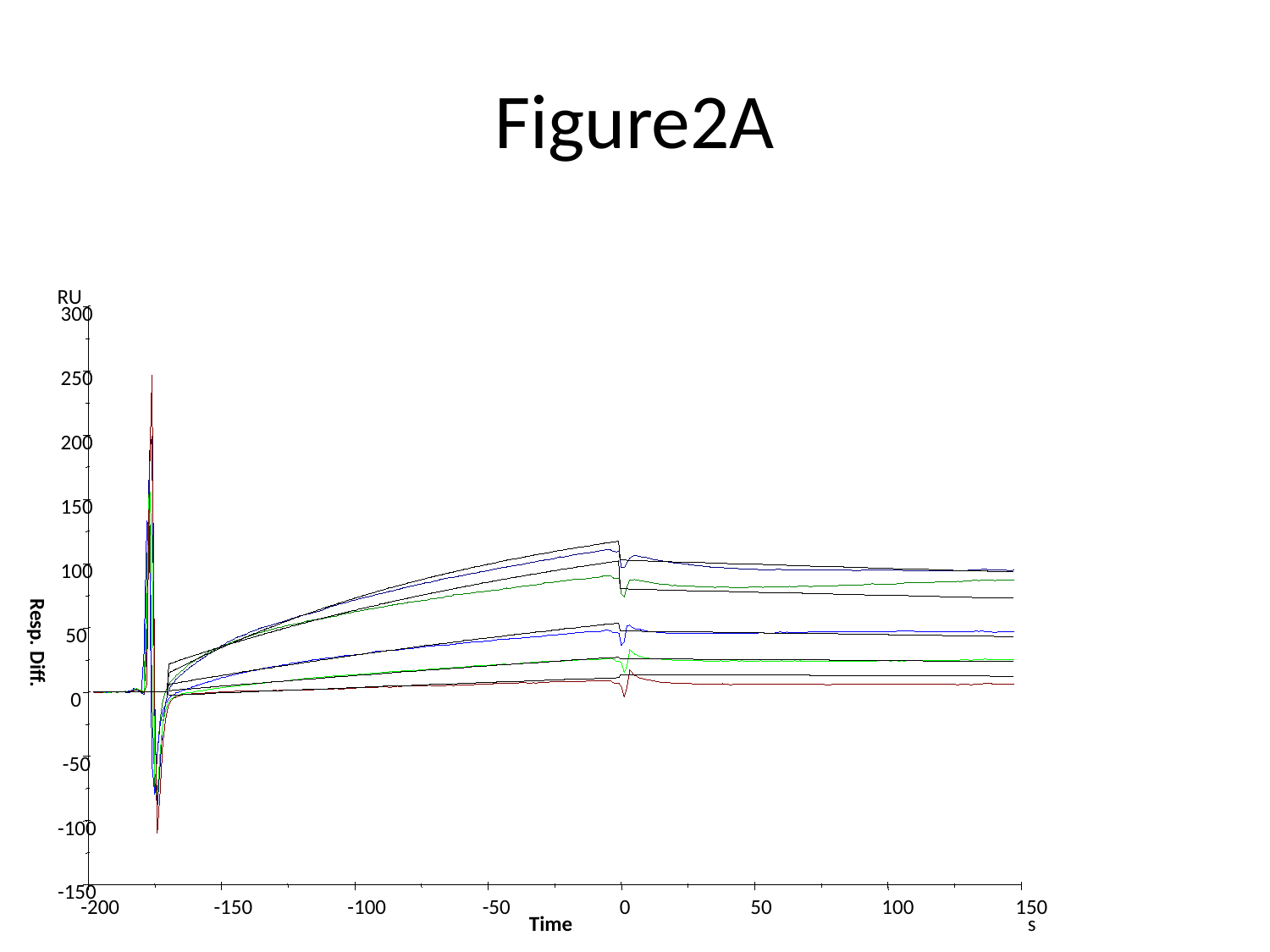

# Figure2A
RU
300
250
200
150
100
50
Resp. Diff.
0
-50
-100
-150
-200
-150
-100
-50
0
50
100
150
Time
s

## Slide 4
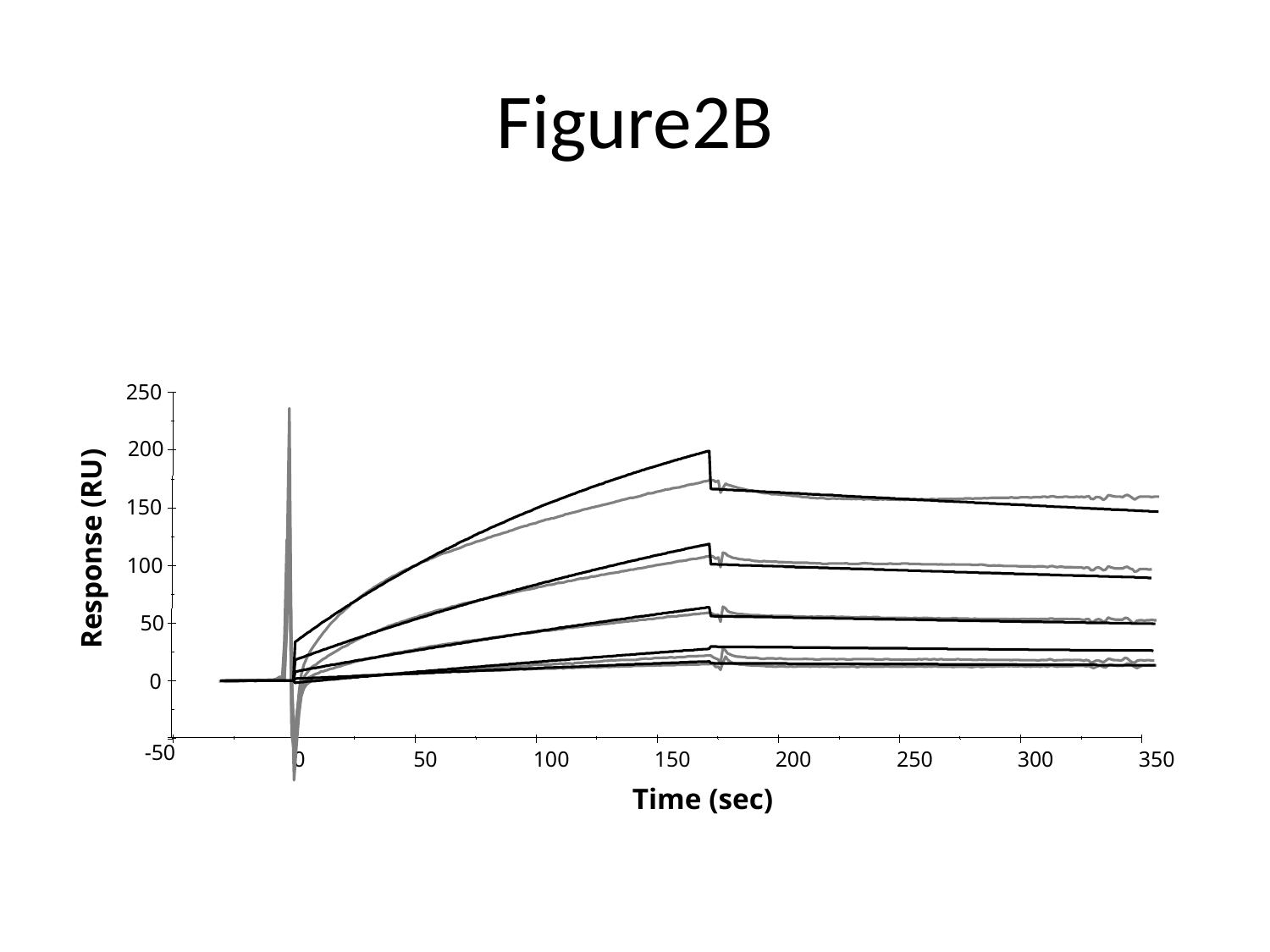

# Figure2B
250
200
150
Response (RU)
100
50
0
-50
0
50
100
150
200
250
300
350
Time (sec)

## Slide 5
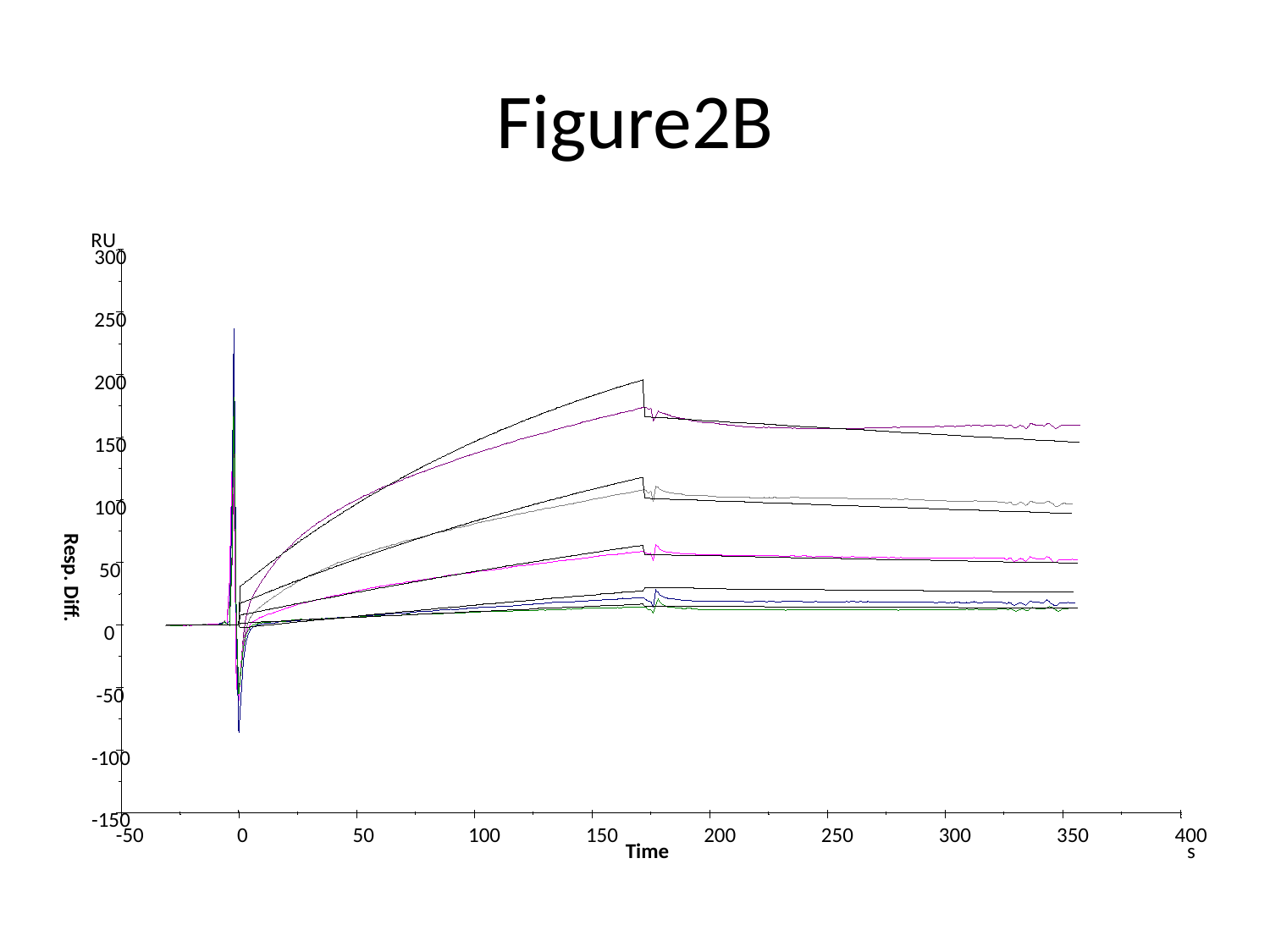

# Figure2B
RU
300
250
200
150
100
50
Resp. Diff.
0
-50
-100
-150
-50
0
50
100
150
200
250
300
350
400
Time
s

## Slide 6
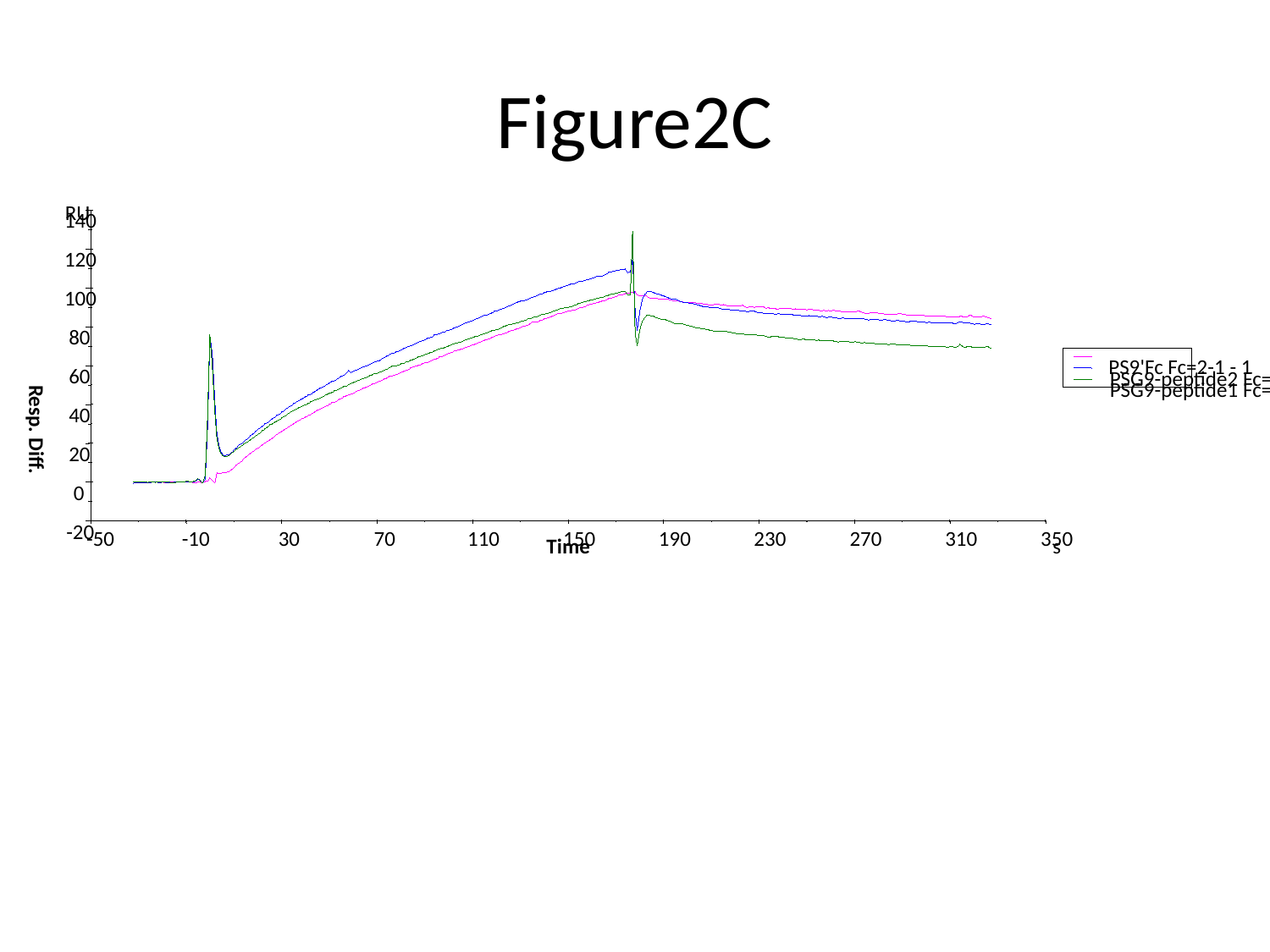

# Figure2C
RU
140
120
100
80
PS9'Fc Fc=2-1 - 1
60
PSG9-peptide2 Fc=2-1 - 1
PSG9-peptide1 Fc=2-1 - 1
40
Resp. Diff.
20
0
-20
-50
-10
30
70
110
150
190
230
270
310
350
Time
s

## Slide 7
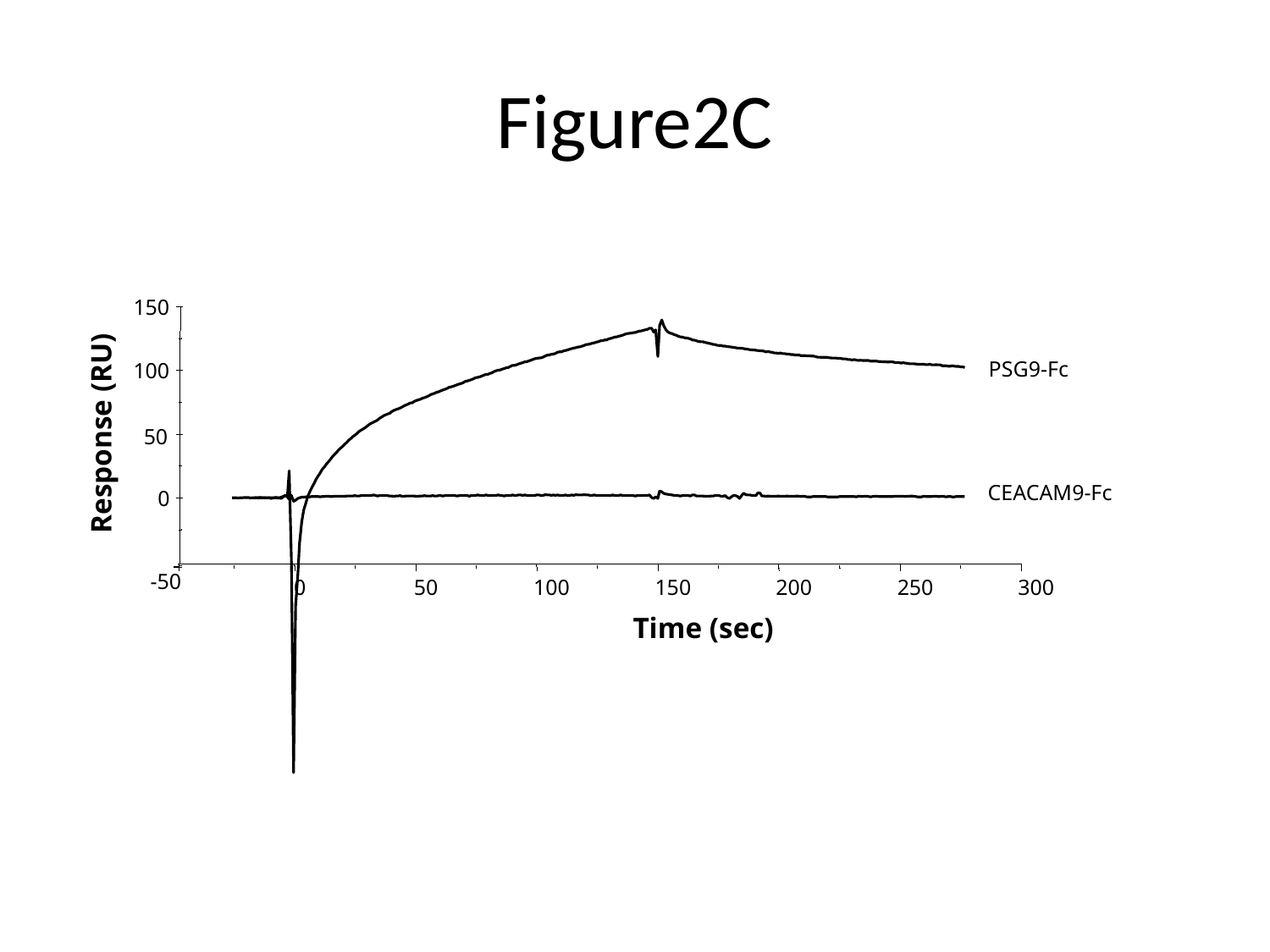

# Figure2C
150
PSG9-Fc
100
Response (RU)
50
CEACAM9-Fc
0
-50
0
50
100
150
200
250
300
Time (sec)
