# Supplementary material for: Correction: PSG9 Stimulates Increase in FoxP3+ Regulatory T-Cells through the TGF-β1 Pathway
Source: PLoS One. 2017 Apr 6;12(4):e0175636. doi: 10.1371/journal.pone.0175636 (PMC5383368; doi:10.1371/journal.pone.0175636)
Supplement: S2 File — (PDF) [file pone.0175636.s002.pdf]

RESEARCH ARTICLE

PSG9 Stimulates Increase in FoxP3+ Regulatory T-Cells through the TGF-β1 Pathway

Karlie Jones[1☯], Angela Ballesteros[2☯], Margaret Mentink-Kane[3], James Warren[3], Shemona Rattila[3], Harry Malech[1], Elizabeth Kang[1], Gabriela Dveksler[3]*

1 National Institute of Allergy and Infectious Diseases, NIH, Bethesda, Maryland, United States of America, **2** National Institute on Deafness and other Communication Disorders, NIH, Bethesda, Maryland, United States of America, **3** Department of Pathology, USUHS, Bethesda, Maryland, 20814, United States of America

☯ These authors contributed equally to this work.
* gabriela.dveksler@usuhs.edu

Check for updates

OPEN ACCESS

Citation: Jones K, Ballesteros A, Mentink-Kane M, Warren J, Rattila S, Malech H, et al. (2016) PSG9 Stimulates Increase in FoxP3+ Regulatory T-Cells through the TGF-β1 Pathway. PLoS ONE 11(7): e0158050. doi:10.1371/journal.pone.0158050

Editor: Dat Quoc Tran, The University of Texas Medical School at Houston, UNITED STATES

Received: February 9, 2016

Accepted: June 9, 2016

Published: July 7, 2016

Copyright: This is an open access article, free of all copyright, and may be freely reproduced, distributed, transmitted, modified, built upon, or otherwise used by anyone for any lawful purpose. The work is made available under the Creative Commons CC0 public domain dedication.

Data Availability Statement: FACS data files are stored at (https://figshare.com/s/dea1406c002c1d6ea7d8). All other relevant data are within the paper and its Supporting Information files.

Funding: This work was supported by the USUHS intramural award R0742495 to GD and the Intramural Research Program of the National Institute of Allergy and Infectious Diseases, National Institutes of Health (NIH) under intramural project number Z-A1-00-0989 supported the research reported in this publication. The funders

Abstract

The pregnancy-specific glycoproteins (PSGs) are a family of proteins secreted by the syncytiotrophoblast of the placenta and are the most abundant trophoblastic proteins in maternal blood during the third trimester. The human PSG family consists of 10 protein-coding genes, some of which have a possible role in maintaining maternal immune tolerance to the fetus. PSG9 was reported as a potential predictive biomarker of pre-eclampsia, a serious complication of pregnancy that has been related to immunological dysfunction at the fetal-maternal interface. Therefore, we hypothesized that PSG9 may have an immunoregulatory role during pregnancy. We found that PSG9 binds to LAP and activates the latent form of TGF-β1. In addition, PSG9 induces the secretion of TGF-β1 from macrophages but not from CD4+ T-cells. TGF-β1 is required for the *ex vivo* differentiation of regulatory T-cells and, consistent with the ability of PSG9 to activate this cytokine, we observed that PSG9 induces the differentiation of FoxP3+ regulatory T-cells from naïve murine and human T-cells. Cytokines that are associated with inflammatory responses were also reduced in the supernatants of T-cells treated with PSG9, suggesting that PSG9, through its activation of TGFβ-1, could be a potent inducer of immune tolerance.

Introduction

Pregnancy specific-glycoproteins (PSGs) are secreted by the placental syncytiotrophoblast from the time of syncytia formation in the blastocyst until term [1,2]. Human PSGs levels have been detected in serum as early as 3 days post fertilization and through the course of pregnancy, reaching concentrations of approximately 200 μg/ml [3]. Several findings are consistent with a role for human PSGs in the modulation of maternal immune responses during pregnancy [4–6]. Depressed PSG levels are also associated with adverse pregnancy outcomes including fetal growth retardation and preterm delivery, suggesting the importance of PSGs for successful pregnancy [7–9].

had no role in study design, data collection and analysis, decision to publish, or preparation of the manuscript.

Competing interests: The authors have declared that no competing interests exist.

There are ten human PSG genes (named PSG1-9, and 11) clustered on chromosome 19q13.1–13.3 [10–13]. Human PSGs are comprised of a leader peptide followed by one N-terminal immunoglobulin (Ig) variable region-like domain (N-domain) and two or three Ig constant region-like domains (A1, A2 and B2 domains)[14]. There is pronounced disparity in expression levels between different members of the family and, despite having significant sequence similarity, whether expansion of this gene family reflects selection for increased gene dosage or for diversification of function, remains unknown [15,16].

The study of PSG9 is of particular interest as its levels have been found by mass spectrometry to differ at 15-weeks' gestation between women diagnosed with early-onset preeclampsia and healthy controls [17]. Some PSGs, including PSG1, have been implicated in the induction of transforming growth factor beta-1 (TGF-β1), a cytokine essential to suppression of inflammatory T-cells and important for differentiation of tolerance inducing $CD4^+CD25^+FoxP3^+$ regulatory T-cells [18,19]. PSG9 shares significant sequence homology with PSG1's N- and B2-domains, which are crucial to PSG1's ability to induce the secretion and activation of latent TGF-β1 (Fig 1A). Because PSG9 seems to play a role in the onset of pre-eclampsia and shares homology with PSG1, we hypothesized that PSG9 is important to the induction of immune tolerance during pregnancy. Treatment of both human and murine naive $CD4^+$ T-cells with PSG9 increased the number of $FoxP3^+$ regulatory T-cells by increasing FoxP3 expression at the protein and mRNA levels. This effect was a direct result of activation of TGF-β1 as a TGF-β1 specific receptor inhibitor prevented the increase in FoxP3 expression. We also observed a significant increase in $CD4^+LAP^+FoxP3^-$ T-cells, which have been previously determined to have regulatory function [20]. In addition, PSG9 reduced the secretion of several pro-inflammatory cytokines and chemokines by $CD4^+$ T-cells. The results presented here bring us one step closer to understanding the role of PSGs in the regulation of the immune response during pregnancy, and suggests the possible therapeutic value of PSG9 for treatment of diseases resulting from the breakdown of immune tolerance.

Materials and Methods

Protein production and purification

The PSG9 cDNA encoding the leader peptide, N, A1, A2 and B2 domains (NCBI reference sequence NM_002784) was subcloned into the pFuse-IgG1 e3-Fc1 vector (Invivogen, San Diego, CA, USA) resulting in the in-frame addition of the hinge region, CH2 and CH3 domains (Fc tag) of the mutated IgG_1 heavy chain. A single cell clone of stably transfected Chinese hamster ovary (CHO)-K1 cells expressing PSG9-Fc was obtained following selection with 250 μg/ml of Zeocin. PSG9-Fc was purified from the supernatant of the PSG9-Fc expressing cell line which was grown on a C2003 hollow fiber cell culture cartridge (FiberCell Systems, Frederick, MD, USA). Production and purification of the control proteins CEACAM9-Fc and FLAG-Fc secreted from stable CHO cells was performed using the same conditions to the ones described for PSG9-Fc and were previously reported [18,21]. CEACAM9-Fc, FLAG-Fc, and medium only gave the same response in all assays performed and both proteins were used as experimental controls interchangeably. Equimolar protein concentrations of PSG9 and control proteins were employed in most experiments unless indicated otherwise. All recombinant proteins generated for the studies described in this manuscript were purified on protein A columns in an AKTA-Prime system (GE Healthcare). Elution fractions containing the protein were pooled, concentrated and buffer exchanged with PBS in an Amicon Ultra-10K centrifugal filter unit (Millipore). For quantitation purposes, the purified proteins were separated on 4–20% NuPAGE Bis-Tris gels (Invitrogen, Grand Island, NY, USA) at different dilutions alongside known concentrations of BSA, used as standards (Thermo Scientific, Waltham, MA,

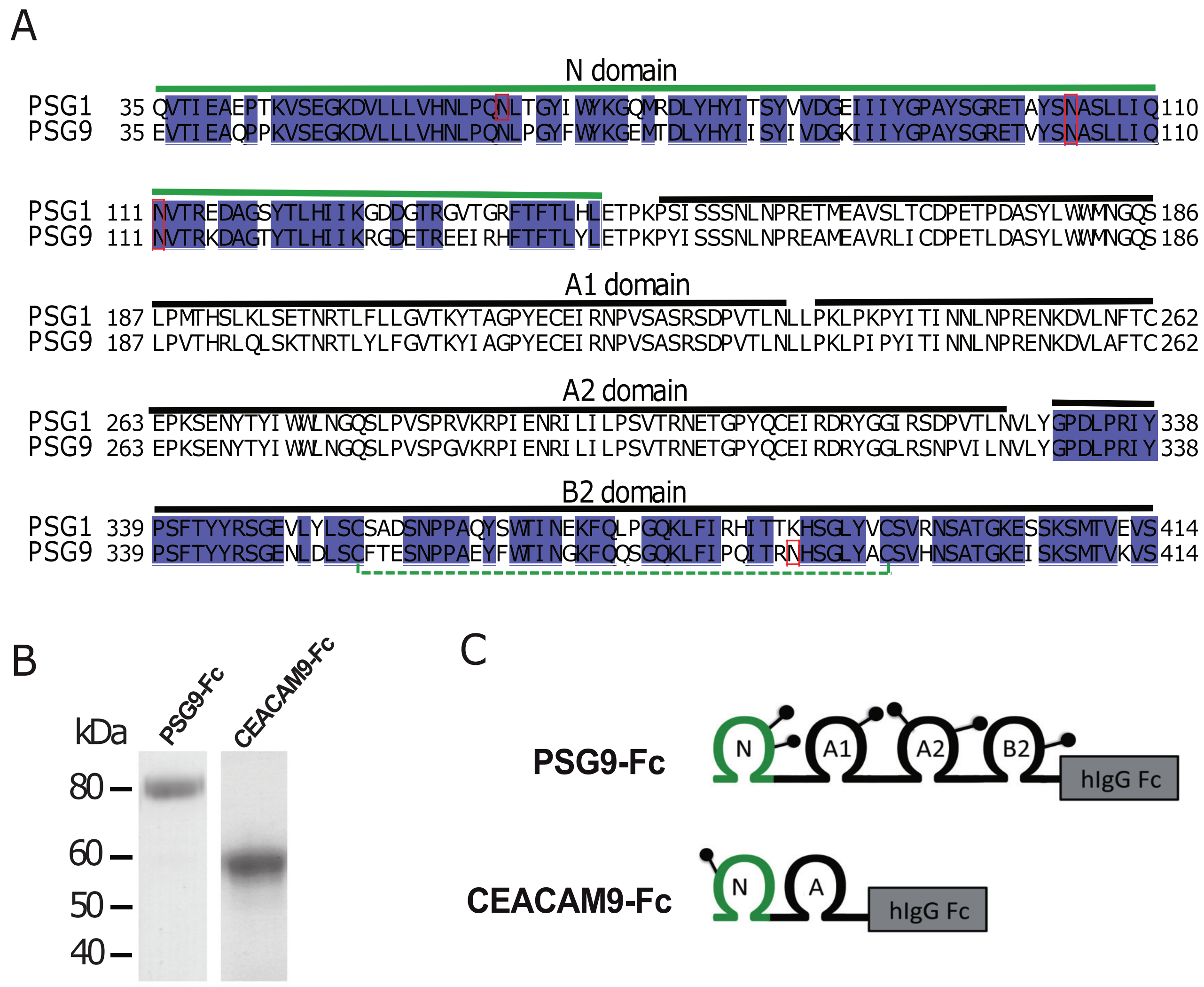

Fig 1. Comparison of PSG1 and PSG9 sequences and depiction of proteins used in the studies. (A) Sequence alignment of human PSG1 and PSG9 (residues 35–415) highlighting the N and B2 domains sequence identity. Sequences corresponding to A1 and A2 domains are also indicated. Potential N-glycosylation sites are boxed in red and the disulfide bond in the B2 domain is marked with a dotted green line. (B) Purified PSG9-Fc and CEACAM9-Fc proteins produced in CHO-K1 cells were loaded into a 4–20% NuPAGE gel. The gel was stained with GelCode Blue to visualize the proteins. C. Schematic representation of the proteins shown in B. The N-terminal IgV domain and the IgC type domains (A1, A2 and B) are shown. Potential N-linked glycosylation sites are indicated as lollipops and the position of Fc protein tag is represented as a box.

doi:10.1371/journal.pone.0158050.g001

USA). After staining with GelCode Blue (Thermo Scientific), the proteins were quantitated by densitometry.

Surface Plasmon Resonance assays

Protein-protein interactions were analyzed by the SPR technique using a Biacore 3000 instrument (GE Healthcare). The experiments were performed as described in [19]. Recombinant human LAP and human latent TGF-β_1, obtained from R&D Systems, Inc (Minneapolis, MN, USA [catalog no. 246-LP, carrier-free and 299-LT, respectively]), were coupled via a standard amine-coupling procedure to the flow cells of a CM5 sensor chip until a level of 1000 resonance units (RU) was reached. A control cell was prepared in the same way but without

protein. Tested proteins were injected separately into the flow cell using a flow rate of 10 μl/min at 25°C. Each interaction was analyzed at least 3 times with different protein preparations. Several analyte PSG9 concentrations (6, 4, 2, 1, 0.5 and 0.25 μM) were injected during the association phase for 3 minutes with HBS-EP (0.01 M HEPES pH 7.4, 0.15 M NaCl, 3 mM EDTA and 0.005% v/v Surfactant P20) as the running buffer. The dissociation phase, initiated by passage of HBS-EP alone, was carried out over a period of 2 minutes. The chip surfaces were regenerated by a 60 s injection of 10 mM Glycine-HCl pH 2.0. Kinetic data were analyzed using BIAevaluation software v4.1.1. The association (K_a) and dissociation (K_d) constants were calculated assuming 1:1 Langmuir binding model. All binding curves were corrected for background and bulk refractive index contribution by subtraction of the reference flow cells.

PSG9-Fc was incubated with the LSKL peptide (GenScript USA Inc.) or a control peptide (SLLK) for inhibition studies. PSG9 at 2 μM was mixed with 10 or 100 fold concentration of peptides for 1 h at 37°C before injection at 10 μl/min over a biosensor chip with immobilized LAP. LAP was coupled via a standard amine-coupling procedure to the flow cell of a CM5 sensor chip until a level of 2000 resonance units was reached. A reference cell was prepared in the same way but without protein. The association and dissociation phases were performed as described above. All binding curves were corrected for background and bulk refractive index contribution by subtraction of the reference flow cell.

Detection of active TGF-β1 and inhibition of TGF-β receptor I

Active TGF-β1 was detected in a cell-free system as previously described [18]. Briefly, 50 ng/mL of SLC (R&D Systems, Inc.) was incubated with PSG9-Fc or control protein at the concentrations indicated in the Figure Legends at 37°C for 1 h in a final volume of 0.1 mL PBS in siliconized tubes. The samples were transferred to a 96-well Nunc Maxisorb plate that had been blocked with 0.5% BSA following an overnight coating step with anti- TGF-β1 (R&D Systems, Inc). After 2 h, the plates were washed and the presence of active TGF-β1 was detected with a biotinylated antibody specific for mature TGF-β1 from the DuoSet ELISA kit (R&D Systems, Inc). The presence of bioactive TGF- β was also determined using the TGF-β responsive PAI-1 luciferase reporter mink lung epithelial cell line (MLEC) provided by Dr. D. Rifkin, as previously described [22]. In brief, 1.6 x 10^4 MLEC cells were seeded onto a 96-well plate in serum-free DMEM with 0.1% ITS (Sigma-Aldrich). After 3 h, the MLECs were treated with either DMEM-0.1% ITS (medium control) or different concentrations of PSG9-Fc or CEACAM9-Fc that had been incubated at 37°C for 1 h with 50 ng/mL SLC. After 18 h the cells were rinsed with PBS, lysed with Promega Passive Lysis Buffer (#E1941) and the lysate analyzed on a luminometer (Promega's GloMax) following addition of the luciferase substrate (#E1501). Recombinant TGF-β1 was also added to the MLECs to generate a dose-response curve from which the luciferase activity could be interpolated.

When indicated, 5 μM SB431542, a specific inhibitor of the TGF-β receptor I, also known as ALK5, was added to the cells for at least 1 hour prior and kept during the duration of the experiment. A treatment group with vehicle (DMSO) was also included.

Murine and Human Cell Culture Conditions to study T_{regs} conversion

For the study of conversion of naïve $CD4^+$ T-cells into T_{regs}, spleens were collected from euthanized 12 week old FoxP3-IRES-GFP knock-in mice on C57BL/6 background provided by Dr. Warren Strober (National Institutes of Health) or from healthy 12 week old C57BL/6 mice. Spleens were crushed and passed through a 100 μm cell strainer (Corning, Manassas, VA) and collected in PBS. Cells were treated with ACK lysing buffer (Quality Biological, Gaithersburg, MD) to remove red blood cells. $CD4^+CD25^-$ T-cells were isolated from splenic cells using a

mouse $CD4^+CD25^+$ Regulatory T-Cell Isolation Kit (Miltenyi, Gaithersburg, MD) and were cultured in 24 well plates at a concentration of $3x10^5$ cells/ml per well. Cells were stimulated with CD3/CD28 T-cell activator Dynabeads (Life Technologies, Grand Island, NY) and were incubated with 50 ng/ml of recombinant human IL-2 (Peprotech) in IMDM (Life Technologies, Grand Island, NY) with 10% fetal bovine serum (Atlanta Biologicals, Flowery Branch, GA) and 100 μg/ml Penicillin/Streptomycin (Quality Biological, Gaithersburg, MD) in the presence of the indicated recombinant proteins.

Human peripheral blood was collected from healthy volunteers after informed consent on an IRB approved protocol. Peripheral blood mononuclear cells (PBMC) were prepared by centrifugation over lymphocyte separation medium (MP Biomedicals, Solon, OH) and contaminating red blood cells lysed with ACK lysis buffer (Quality Biological, Gaithersburg, MD). $CD4^+CD25^-$ T-cells were isolated using the human $CD4^+CD25^+$ Regulatory T-Cell Isolation Kit (Miltenyi, Gaithersburg, MD). Cells were cultured as stated previously in RPMI (Life Technologies, Grand Island, NY) with 10% fetal bovine serum (Atlanta Biologicals, Flowery Branch, GA) and 100 μg/ml Penicillin/Streptomycin (Quality Biological, Gaithersburg, MD) in the presence of the indicated recombinant proteins. Fold change was used to quantify human data as volunteers differed in age, health, and gender affecting baseline FoxP3 expression.

RNA extraction and real-time PCR

Total RNA was collected from mouse T-cells cultured as described above. RNA was isolated using the RNAeasy Plus Mini Kit (Qiagen, Germantown, MD). cDNA was synthesized from 100 ng of total RNA using the SuperScript VILO cDNA synthesis kit (Life Technologies, Grand Island, NY) according to the manufacturer's recommendation. RT-PCR for FoxP3, IL-2, IL-6 and GAPDH transcripts was performed on a 7500 Real-Time PCR system (Applied Biosystems, Foster City, CA). Primers used are as follows: FoxP3 FAM-MGB (Mm00475162_m1), IL-2 FAM-MGB (Mm00434256_m1), IL-6 FAM-MGB (Mm00446190_m1) and GAPDH VIC-MGB (4352339E) (Life Technologies, Grand Island, NY). All PCRs were performed using TaqMan Universal PCR Master Mix (Applied Biosystems, Foster City, CA). Relative gene expressions were normalized to GAPDH.

Flow Cytometric Analysis

Seventy- two hours after plating, the cells were washed and resuspended in serum and azide free PBS (Life Technologies, Carlsbad, CA) and were stained with the eFluor 780 Viability dye (eBioscience, San Diego, CA) for 30 minutes at 4°C. Following washing, the cells were incubated with anti-mouse CD4-FITC (RM4-5) and CD25-APC (PC61.5) or with anti-human CD4-FITC (OKT4) (eBioscience, San Diego, CA) and CD25-PE (BD Biosciences, San Jose, CA) for 30 minutes at room temperature. A labeled isotype-matched control antibody was used as a control in these experiments. Cells were fixed and permeabilized using the FoxP3 Staining Buffer Set (eBioscience, San Diego, CA), and then stained with anti-mouse FoxP3-PE (FJK-16s), anti-human FoxP3-APC (236A/E7) (eBioscience, San Diego, CA) or anti-human LAP-PerCP-Cy5.5 (BD Biosciences, San Jose, CA). Human samples were gated for CD4 expression prior to gating for FoxP3 expression. Samples were run on a benchtop BD FACSCanto flow cytometer (BD Biosciences, San Diego, CA) and analysis was performed using FlowJo FACS analysis software (FlowJo LLC, Ashland, OR).

Cytokine and Chemokine Measurements

Secretion of TGF-β1 by the murine macrophage cell line RAW264.7 was determined as previously described [23]. To determine whether the observed increase in TGF-β1 secretion in

RAW264.7 cells was mediated by a TGF-β1 positive feedback loop, cells were treated with PSG9N or the control protein in the presence of SB431542 or vehicle. The effects of PSG9 in the secretion of IL-2 and TGF-β1 in $CD4^+$ T-cells, were studied in $CD4^+$ T-cells isolated from the spleen of C57BL/6 mice using $CD4^+$ T Cell Biotin Antibody Cocktail and Anti-Biotin Microbeads (Miltenyi, Gaithersburg, MD). Isolated $CD4^+$ T-cells were seeded in 96-wells plates at 10^5 cells/well and activated with 2.5 μl of CD3/CD28 T-cell activator Dynabeads (Life Technologies) in a 150 μl final volume of IMDM supplemented with 10% fetal clone III serum (HyClone, GE Healthcare), 50 ng/ml IL-2 and Penicillin/Streptomycin (to measure TGF-β1) or in IMDM with 10% fetal bovine serum and Penicillin/Streptomycin in the presence of the indicated recombinant proteins. Supernatants were collected at the indicated times and IL-2 and TGF-β1 (following acid activation) were measured by ELISA following the manufacturer's recommendations (R&D Systems, Inc). All treatments were performed in triplicate and three independent experiments were completed.

Cytokine panels were performed on supernatants from three-day cell culture of both mouse and human T-cells as described in the cell culture assays to study T_{reg} conversion methods as above. Cytokine and chemokine concentrations were measured using Luminex cytokine/chemokine multiplex immunoassay kits (R&D Systems, Minneapolis, MN) with the BioPlex System (Bio-Rad, Hercules, CA). Due to varying basal levels of cytokines in human supernatants from different donors, human cytokines levels are shown as fold change rather than absolute concentration.

Statistical analysis

Prism software (GraphPad Software, La Jolla. CA) was used for all statistical analysis where appropriate. Data were analyzed using One-Way ANOVA or Two-Way ANOVA followed by Bonferroni post hoc analysis or the Student's *t* test to compare experimental groups. The type of test used for each data set is specified in the figure legend and all data are representative of at least three independent experiments. Unless otherwise indicated, graphical data represent the mean ± SEM.

Results

PSG9-Fc binds to the SLC and LAP of TGFβ-1

TGF-β1 is widely expressed in leukocytes and stromal cells, but unlike other cytokines that are produced in biologically active forms, TGF-β1 must undergo an activation process to mediate its effects. TGF-β1 is translated as an inactive pre-pro-TGF-β1 consisting of a signal peptide, the 250-residue latency associated peptide (LAP) and the 110-residue mature TGF-β1 peptide [24]. This latent form of TGF-β1, also known as the small latent complex (SLC), cannot bind to its receptors and signal until proteolytic cleavage or a conformational change in LAP exposes the mature TGF-β1 polypeptide [25]. To determine whether PSG9 interacts with TGF-β1, we generated recombinant PSG9 consisting of the leader peptide, the N, A1, A2 and B2 domains and the cytoplasmic tail followed by the Fc tag, which consists of the human IgG_1 hinge, CH2 and CH3 domains as described under the materials and methods section. The recombinant protein, designated PSG9-Fc, has an approximate molecular mass of 80 kDa when separated on SDS-PAGE (Fig 1B). As a negative control protein for our studies, we used Fc-tagged CEACAM-9, which, like PSGs, is a member of the CEA family. The CEACAM9-Fc was generated and purified under identical conditions as PSG9-Fc [18] (Fig 1B and 1C).

We analyzed whether PSG9 can bind to the SLC using Surface Plasmon Resonance (SPR) analysis and defined the kinetics of the interaction between these proteins. SPR was also utilized to determine if PSG9 binds to LAP in the absence of the mature TGF-β1. In order to characterize the kinetics of the PSG9 interaction with LAP and the SLC of TGF-β1, we injected

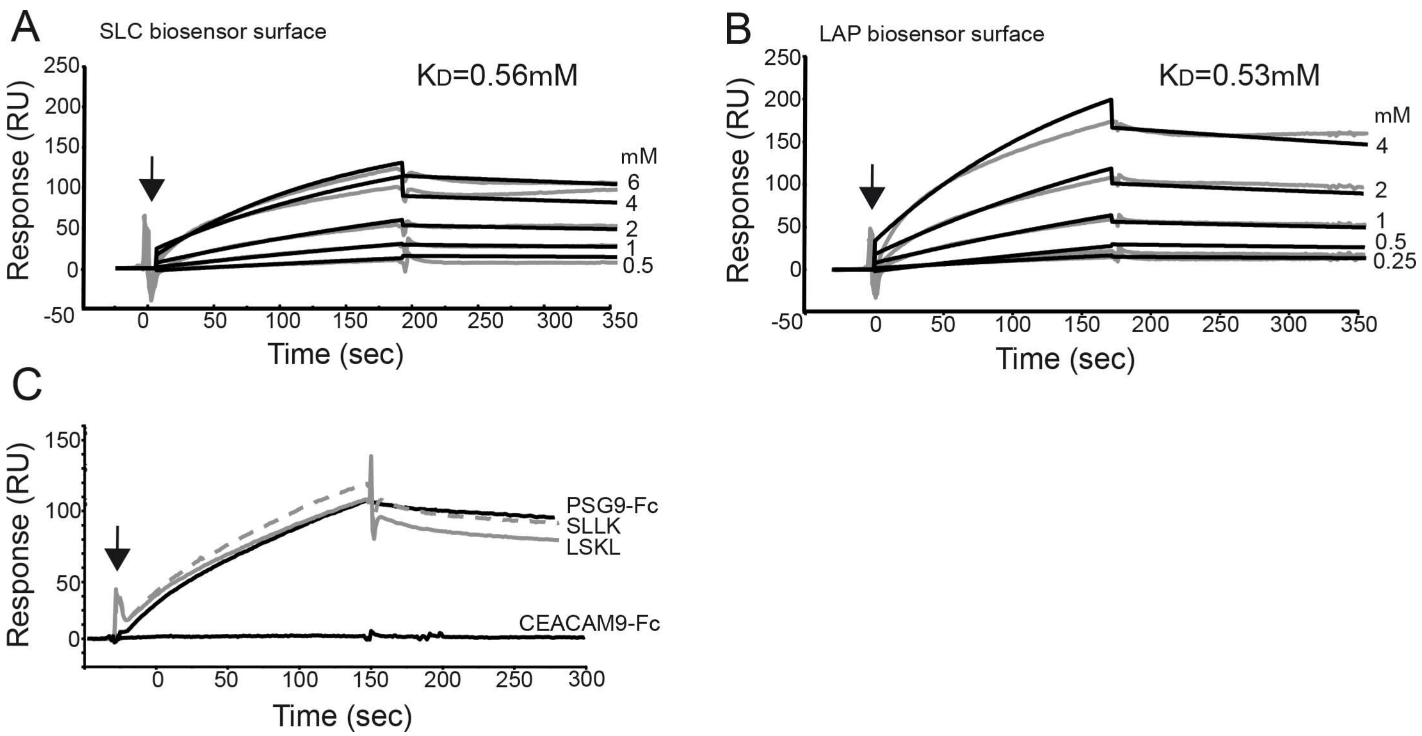

Fig 2. PSG9 interacts with SLC and LAP of TGF-β1. Purified PSG9-Fc protein was injected at a concentration range from 0.25 to 6 μM over a CM5 sensor chip with immobilized SLC (A) and LAP (B) of TGF-β1. PSG9-Fc injection point is indicated with an arrow and the KD values for each interaction are shown. SPR sensograms for each response are shown as gray lines while fit analyses are shown as black lines. C. PSG9-Fc at 2 μM was incubated with the LSKL peptide antagonist of thrombospondin-1 or a control peptide (SLLK) before injection over a CM5 sensor chip with immobilized LAP. Sensogram for CEACAM9 injected over the LAP biosensor is also shown as a control. RU, response units.

doi:10.1371/journal.pone.0158050.g002

serial dilutions of PSG9 ranging from 0.25 to 6 μM over a blank, SLC or LAP biosensor surface and measure in real time the association (Ka) and dissociation (Kd) rates and binding constant (KD) for each interaction (Fig 2A and 2B and Table 1). PSG9 presented similar binding constants for the interaction with LAP and the SLC of TGF-β1, 0.53 and 0.56 μM respectively, suggesting a direct interaction with LAP that is not affected by the presence of the active TFG-β1 peptide. As expected, the control protein, CEACAM-9, did not bind to LAP or the SLC of TGF-β1 (Fig 2C).

The best characterized activator of TGF-β1 is the matricellular protein TSP-1, which is contained in platelet α-granules and participates in wound healing and fibrosis [26]. Specific sequences in the type I repeats of TSP-1 interact with amino acids 54 to 57 (LSKL) at the N-terminus of LAP, altering the folding of LAP in relation to mature TGF-β1 and unmasking the receptor binding sites [27,28]. To determine whether binding of PSG9 to LAP occurs via

Table 1. Kinetic analysis of PSG9-Fc interaction with human SLC and LAP of TGF-β_1.

LAP	KD μM	K_a (mean ± SD) $M^{-1}s^{-1}$	k_d (mean ± SD) s^{-1}
PSG9-Fc	0.53	(1.30 ± 0.29) x 10^3	(0.68 ± 0.09) x 10^{-3}
**Latent TGF-β1**	**KD μM**	**K_a (mean ± SD) $M^{-1}s^{-1}$**	**k_d (mean ± SD) s^{-1}**
PSG9-Fc	0.56	(1.15 ± 0.24) x 10^3	(0.65 ± 0.09) x 10^{-3}

The kinetic data shown in Fig 2 for the interaction of PSG9-Fc with SLC and LAP were fit using a 1:1 Langmuir binding model for the estimation of the association (k_a) and dissociation rates (k_d) and dissociation constant (KD = k_d/k_a).

doi:10.1371/journal.pone.0158050.t001

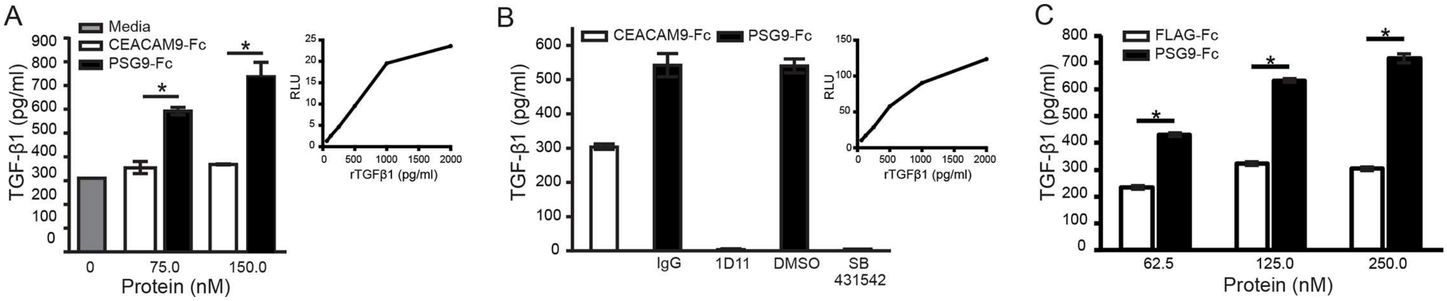

Fig 3. PSG9-Fc activates the SLC of TGF-β. (A) PSG9-Fc or control protein CEACAM9-Fc were incubated at the indicated concentrations with 50ng/mL of the SLC of TGF-β1 in DMEM-0.1% ITS media and added to luciferase reporter cells (MLEC). Media containing the SLC of TGF-β1 in absence of protein was used as a control and designated as medium. The level of mature TGF-β1 in treated MLECs was estimated based on luciferase activity (RLU: relative light units) and is expressed as pg/ml of TGF-β1 following conversion of RLU using rTGF-β1 to generate a standard curve as shown. (B) PSG9-Fc or control protein CEACAM9-Fc were incubated with SLC as indicated in A. Prior to incubation with MLECs, 60 μg/ml of the pan TGF-β neutralizing 1D11 mAb or 5 μM of the TGF-β receptor I kinase inhibitor SB4315422 or the indicated controls were added to the mix. The standard curve generated following incubation of the cells with r TGF-β1 for the conversion of RLU to pg/ml of TGF-β1 is shown (C) PSG9-Fc or the control protein FLAG-Fc, were incubated at increasing concentrations with 50 ng/mL of the SLC of TGF-β1 for 1 h at 37°C. ELISA plate coated with anti- TGF-β1 antibody was used to detect active TGF-β1 levels in the samples as described in Materials and Methods. Mean ± SEM values are shown and Student's *t*-test was employed for statistical analysis. All treatments were performed in triplicate wells with one representative experiment of three independent repeats shown.

doi:10.1371/journal.pone.0158050.g003

amino acids 54–57 in LAP, we performed SPR analysis in the presence of the LSKL peptide or a scrambled control peptide (SLLK). As shown in Fig 2C, pre-incubation of PSG9 with the peptides did not inhibit the interaction between PSG9 and LAP coated on the chip, indicating that PSG9 activates TGF-β1 through a different mechanism than TSP-1.

PSG9 activates the small latent complex of TGFβ-1

To determine if the interaction of PSG9 with SLC results in activation of TGF-β1, we examined the generation of biologically active TGF-β1 using a sensitive and specific quantitative bioassay in which mink lung epithelial cells (MLECs) were stably transfected to express luciferase under the PAI-1 promoter [22]. When the SLC was incubated with PSG9 prior to addition of the MLECs, we observed a dose-dependent increase in luciferase activity, which was not observed upon incubation of the SLC with the control proteins CEACAM9-Fc (Fig 3A). To examine the specificity of the response, we performed similar experiments in the presence of the neutralizing anti- TGF-β1 antibody (1D11) or SB431542, a specific inhibitor of the TGF-β receptor I also known as ALK5. MLECs treated with PSG9 and SLC in the presence of either 1D11 or SB431542 failed to show an increase in luciferase activity while we observed a similar increase in luciferase activity when adding PSG9-Fc in presence of the control IgG or DMSO (Fig 3C). In addition, we observed that PSG9 can dose-dependently activate the SLC in a cell-free system as shown in Fig 3C.

The N-terminal domain of PSG9 induces TGF-β1 in RAW264.7 macrophages but not in CD4+ T-cells

It has been shown previously that the N-terminal domain of PSG1 mediates the ability of PSG1 to induce TGF-β1 secretion in macrophages and that amino acids $L_{41}H_{42}Y_{43}H_{44}$ in the exposed CC' loop of the N-domain of the protein are essential for this activity [23,29]. Because the amino acids LHYH are conserved in PSG9, we tested the capacity of the PSG9 N-terminal domain (PSG9N) to induce TGF-β1 secretion from the RAW264.7 macrophage cell line (Fig 4A). RAW264.7 cells treated with PSG9N-Fc produced TGF-β1 in a dose-dependent manner (Fig 4B). When RAW264.7 cells were treated with PSG9N in the presence of SB-431542, we

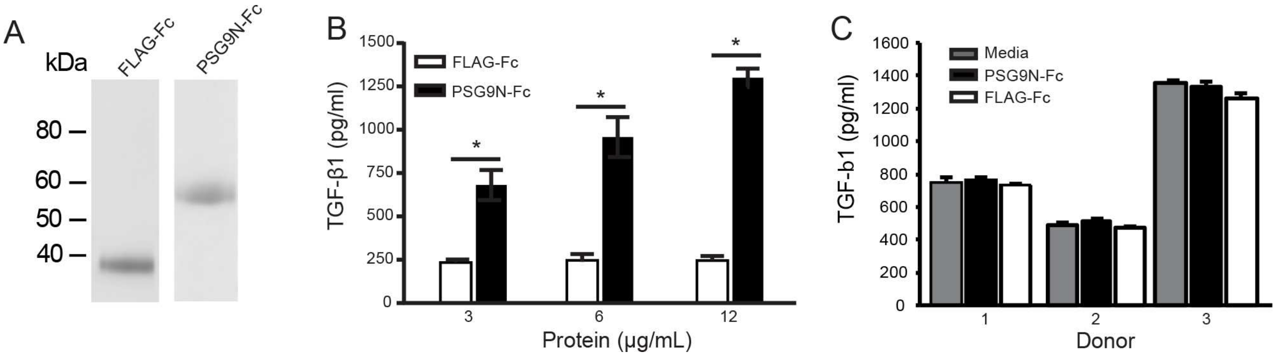

Fig 4. PSG9N induces TGF-β1 secretion from macrophages but does not from $CD4^+$ T-cells. (A) Recombinant PSG9N-Fc and FLAG-Fc produced in CHO-K1 cells were purified on a protein A column and loaded onto a 4–20% NuPAGE gel. The gel was stained with GelCode Blue to visualize the proteins. (B) TGF-β1 secretion following incubation of the macrophages cell line RAW264.7 with increasing concentrations of PSG9N-Fc or control protein. TGF-β1 was measured in the supernatants by ELISA 24 h post-treatment. (C) TGF-β1 secretion of anti-CD3/CD28-activated $CD4^+$ human T-cells isolated from three different donors following incubation with 12 μg/ml of PSG9N-Fc or control protein. TGF-β1 was measured in the supernatants by ELISA 72 h post-treatment. Mean ± SEM values are shown and Student's *t*-test was employed for—statistical analysis.

doi:10.1371/journal.pone.0158050.g004

observed a significant increase in TGF-β1 in the cell supernatant when compared to protein-control treated cells, indicating that the observed increase in TGF-β1 is not due to a positive feedback loop (data shown in S3 File). We then tested whether PSG9N-Fc could induce the secretion of TGF-β1 from activated $CD4^+$ T-cells. To this end, purified mouse splenic $CD4^+$ T-cells were activated with CD3/CD28 T-cell activator Dynabeads and treated with 6 and 12 μg/ml of PSG9N-Fc for 24, 48 and 72 h in the presence of IL-2. There was no difference in the amount of TGF-β1 in the cell supernatant collected from the PSG9N-treated and protein control-treated cells (data not shown). Similar results were obtained upon treatment of $CD4^+$ human T-cells isolated from human peripheral blood of three donors activated with CD3/CD28 T-cell activator Dynabeads in the presence of 12 μg/ml of PSG9N-Fc or control protein and analyzed at 72 h post-treatment (Fig 4C). Therefore, while the N-terminal domain of PSG9 induces the secretion of TGF-β1 from macrophages, it does not increase the secretion of TGF-β1 from activated human $CD4^+$ T-cells.

PSG9 converts naive $CD4^+$ T-cells into $FoxP3^+$ Regulatory T-cells in a TGF-β1 -dependent manner

The TGF-β1 downstream transcription factor SMAD3 mediates Foxp3 induction and IL-2 suppression [30–32]. Therefore TGF-β1 is essential for the maintenance of expression of the transcription factor Foxp3, suppressor function of T_{regs} and survival of peripherally-derived regulatory T-cells [33]. Because PSG9 was able to induce activation of latent TGF-β1 and activated $CD4^+$ T-cells secrete latent TGF-β1, we hypothesized that PSG9 might be sufficient to promote the conversion of naïve $CD4^+$ T-cells into Foxp3-expressing T-cells. To determine this, we isolated naïve ($CD4^+$ $CD62^+$) T-cells from the spleens of Foxp3-IRES-GFP knock-in mice that express a functional GFP only when the Foxp3 gene is transcriptionally active [34]. Treatment of these cells with PSG9 following stimulation with anti-CD3 and anti-CD28 antibodies resulted in an increase of GFP-expression over cells treated with control Flag protein in a dose-dependent manner without affecting cell viability as determined with the eFluor 780 Viability dye (Fig 5A and data not shown). The increase in the percentage of GFP-expressing cells was not observed when the cells were treated with PSG9 in the presence of the TGFβ receptor I inhibitor SB431542 (Fig 5B and 5C), indicating this response was TGF-β1

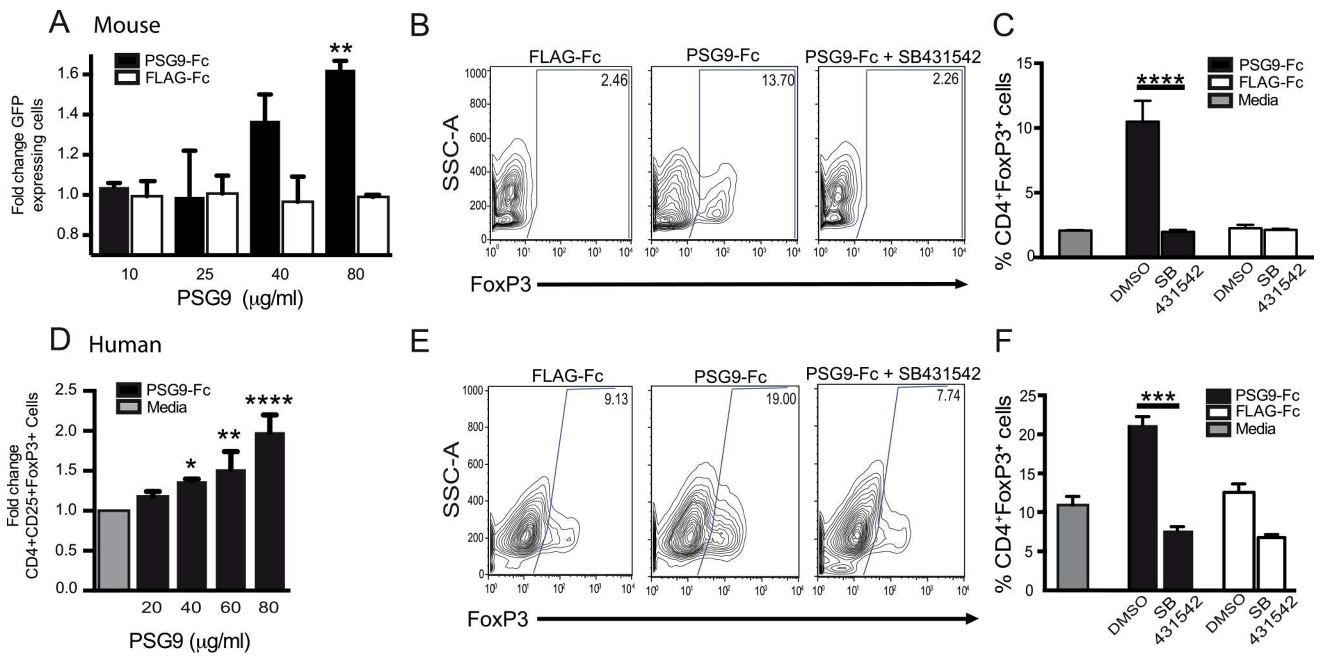

Fig 5. PSG9 converts murine and human naive CD4$^+$ T-cells into FoxP3$^+$ cells in a TGF-β dependent manner. (A) T-cells isolated from the spleens of FoxP3-GFP transgenic mice were stimulated with T-cell activator Dynabeads in the presence of IL-2 and increasing concentrations of PSG9-Fc or FLAG-Fc control and analyzed at 72 h for GFP expression. $P < 0.0005$. (B) Naive mouse CD4$^+$ T-cells were treated with PSG9-Fc or FLAG-Fc in the presence of SB-431542 or vehicle (DMSO) and analyzed as in A. (C) Graphical representation of the flow cytometry data shown in part (B). $P < 0.0001$. D) CD4$^+$ naive T-cells isolated from peripheral blood were stimulated with human T-cell activator Dynabeads in the presence of IL-2 and increasing concentrations of PSG9-Fc or control protein. Analysis was performed 72 h after activation. Cell populations were gated on viable CD4$^+$CD25$^+$ cells and were stained with APC-labeled anti-human FoxP3antibody. $P < 0.0001$. (E) Naive human CD4$^+$ T-cells were treated with PSG9-Fc or FLAG-Fc in the presence of SB-431542 or DMSO, and analyzed at 72 h by flow cytometry for FoxP3 expression. (F) Graphical representation of the flow cytometer data shown in part (E). $P < 0.0005$. Cells were initially gated on viability, followed by gating on CD4 and FoxP3 (human) or GFP expression (mouse). The data shown are representative of at least three independent experiments performed in triplicate. P-values were calculated using one-way ANOVA (B, D and E) and two-way ANOVA (A).

doi:10.1371/journal.pone.0158050.g005

dependent. In addition, we observed an increase in FoxP3 expression at the mRNA level in the PSG9-treated cells when compared with control Flag-treated cells (data not shown). We next examined if PSG9 was also able to increase FoxP3 expression in human T-cells. Human CD4$^+$ T-cells isolated from peripheral blood were incubated with the indicated proteins in the presence of IL-2, and FoxP3 expression was measured by flow cytometry. As observed for naïve mouse T-cells, PSG9 increased FoxP3 expression in human T-cells in a dose-dependent and TGF-β1 specific manner (Fig 5D–5F).

The LAP/TGF-β1 complex is found on the cell membrane of some immune cells, including regulatory T-cells [35,36]. We found that upon treatment with PSG9, human FoxP3$^+$ regulatory T-cells did not show an increase in LAP expression (Fig 6A). Interestingly, we observed an increase in CD4$^+$LAP$^+$FoxP3$^-$ cells, a recently discovered novel subset of regulatory T-cells (Fig 6B) (23). Therefore, PSG9 induces immune tolerance not only through induction of FoxP3 expression in naive CD4+ T-cells but is also able to induce the differentiation of a suppressive LAP$^+$FoxP3$^-$ T-cell subset.

PSG9 inhibits IL-2 expression by CD4$^+$ T-cells

Suppression of IL-2 production by CD4$^+$ T-cells is an important process mediating immune regulation by TGF-β1 [37]. In accordance with the increased availability of active TGF-β1 mediated by PSG9, we found that PSG9 inhibited the secretion of IL-2 by activated murine

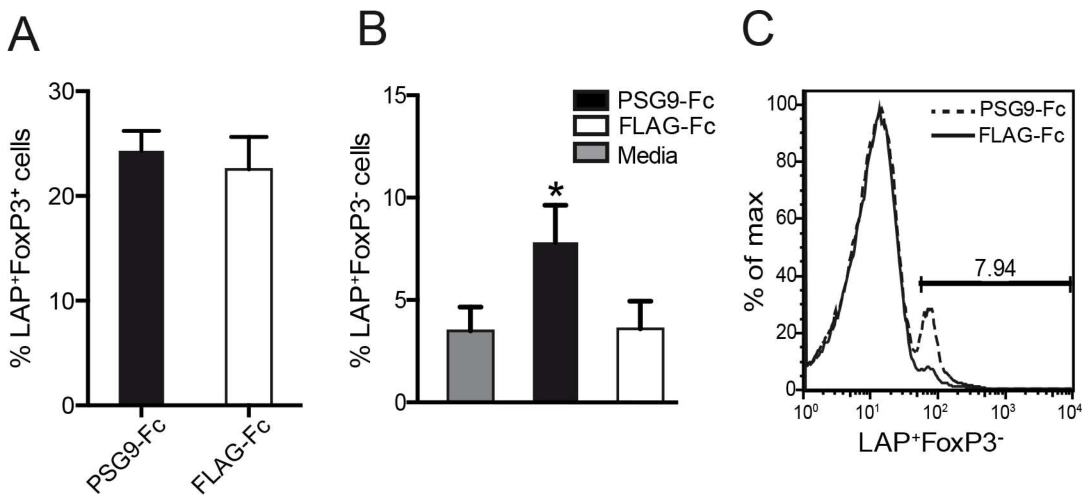

Fig 6. PSG9 induces the expression of a $CD4^+LAP^+FoxP3^-$ regulatory T-cell subset. (A) Human $CD4^+$ naive T-cells were isolated from peripheral blood and plated with human T-cell CD3/CD28 T-cell activator Dynabeads in the presence of IL-2 and PSG9 or FLAG control protein. Expression of $CD4^+FoxP3^+LAP^+$ cells was analyzed at 72 h. (B) Graphical representation of the flow cytometry data shown in part C, $P < 0.05$ (C) PSG9 induces increased LAP expression on the surface of $CD4^+$ $FoxP3^-$ cells. Data shown are representative of at least three independent experiments and P values were calculated using the Student's *t*-test.

doi:10.1371/journal.pone.0158050.g006

$CD4^+$ T-cells measured in culture supernatants at 72 h post treatment (Fig 7A). When these same cells were treated with PSG9 combined with SB431542, IL-2 secretion was unaltered, showing that the PSG9-mediated IL-2 suppression is TGF-β1 dependent (Fig 7B). As shown by qRT-PCR, PSG9 inhibited IL-2 expression at the transcriptional level and it also inhibited the expression of IL-6, a cytokine that inhibits the generation of $FOXP3^+$ T_{regs} cells induced by TGF-β1 (Fig 7C)[38,39].

PSG9 treatment of naive T-cells induces a more tolerogenic cytokine profile

The expression of pro-inflammatory cytokines is a common aspect of autoimmune disorders and is associated with the increased proliferation of cytotoxic T-effector cells, a cell type associated with reduced immune tolerance [40,41]. Aberrant and persistent production of pro-inflammatory cytokines and chemokines can lead to a variety of pregnancy disorders including pre-term birth, fetal growth restriction and preeclampsia and therefore we focused on determining whether PSG9 is able to reduce their expression [42]. We measured cytokine levels in supernatants from naive mouse $CD4^+$ T-cells and human T-cells stimulated *in vitro* using a cytokine/chemokine multiplex immunoassay. We found that after treatment of cells with PSG9, there was a significant reduction in several pro-inflammatory cytokines (Fig 8). All seven of these cytokines were significantly reduced in cell supernatants treated with PSG9. In addition, IL-2 was reduced with PSG9 treatment, correlating with our previous experiments. Finally, we observed that the cytokine IL-9 was significantly increased in PSG9 treated cells (Fig 8A, last panel). IL-9 produced by T_{regs} has been shown to be essential for mast cell recruitment to tolerant tissue and promotes graft tolerance in solid organ transplants [43].

Discussion

TGF-β1 knockout mice have been shown to have reduced survival and excessive inflammatory responses [44]. Additionally, FoxP3 loss-of-function in both humans and mice results in fatal

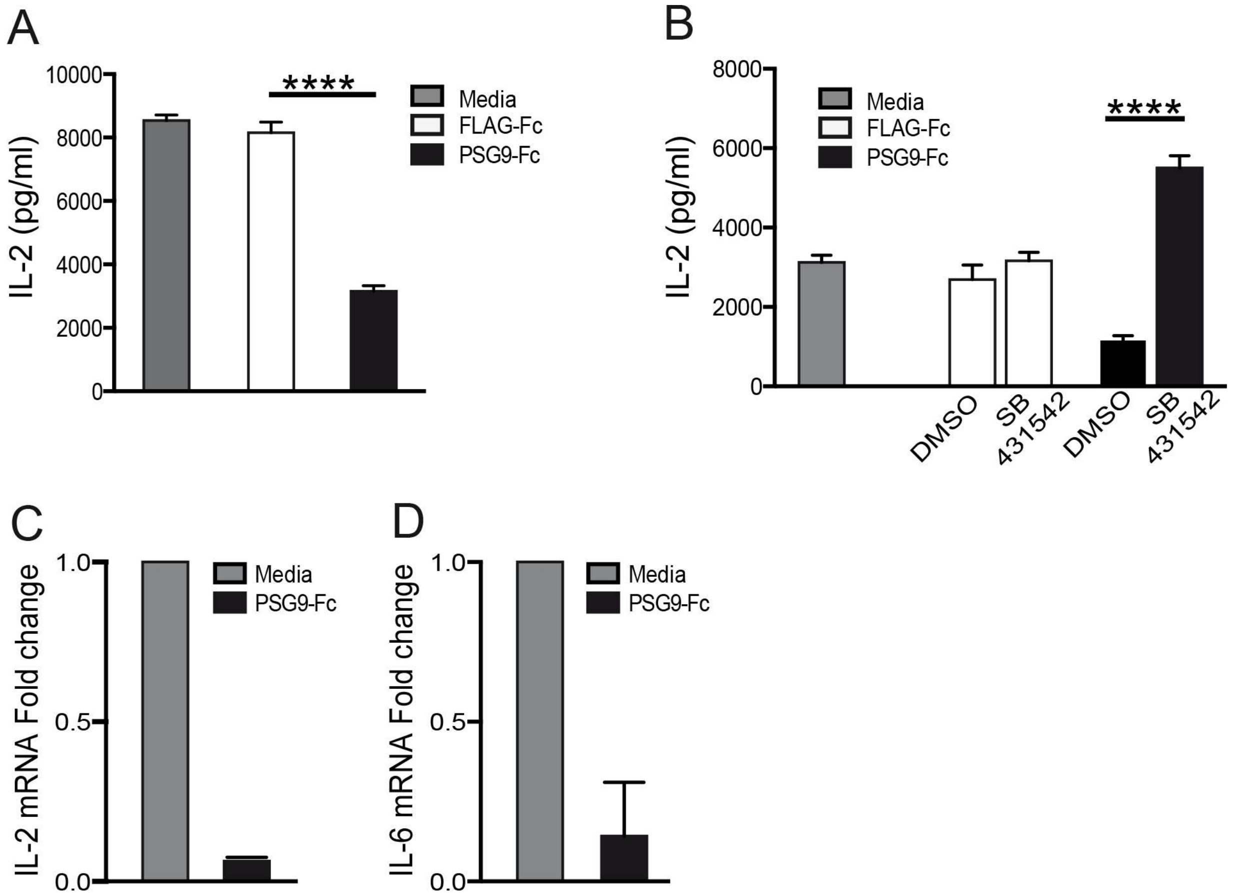

Fig 7. Administration of PSG9 to naive mouse CD4$^+$ T-cells inhibits IL-2 expression. (A) Cells collected from normal C57BL/6 mouse spleens were treated with equimolar concentration of PSG9 or FLAG control protein and activated with mouse CD3/CD28 T-cell activator Dynabeads. IL-2 levels were determined by ELISA 72 h after activation. P value was calculated using the Student's *t*-test, P < 0.0001. (B) Cells were collected, activated, and treated with FLAG or PSG9 as in A in presence of a TGF-β receptor I inhibitor (SB431542) or vehicle (DMSO). IL-2 levels were measured in the supernatant by ELISA 72 h after activation. P value was calculated using one-way ANOVA, P < 0.0001. (C) IL-2 and IL-6 mRNA levels were evaluated by RT-PCR from mouse naive CD4$^+$ T-cells treated with PSG9 or media. P value was calculated using the Student's *t*-test, P < 0.005. Data shown are representative of at least three independent experiments.

doi:10.1371/journal.pone.0158050.g007

systemic immune mediated tissue damage [45–47]. Active TGF-β1 is essential for the differentiation of FoxP3$^+$ regulatory T-cells, which are in turn crucial for maintenance of immune tolerance during pregnancy and prevention of autoimmune reactions [48]. In this study, we describe the interaction of recombinant PSG9 with TGF-β1 and show that this placental protein has the ability to induce regulatory T-cells through a TGF-β-dependent mechanism, suggesting its role as an immune regulator and its potential use as a novel therapeutic agent for pathologies caused by excessive and undesired immune activation.

SPR technology has been successfully used to study the interaction of TGF-β with its cognate receptors and of the mature TGF-β1 polypeptide with LAP [49,50]. We used the same technology to examine the interaction of PSG9 with LAP and the SLC of TGF-β1 and explored the ability of PSG9 to activate latent TGF-β1. We observed that PSG9 directly binds to LAP and to the SLC of TGF-β1, showing similar binding kinetics for both, revealing that the presence of TGF-β1 in the SLC complex does not affect the PSG9-LAP interaction. The SPR experiments reported here also revealed that PSG9 composed of the four domains (N, A1, A2, and B2) and fused to the Fc portion of human IgG1 binds very stably to LAP, with the affinity of

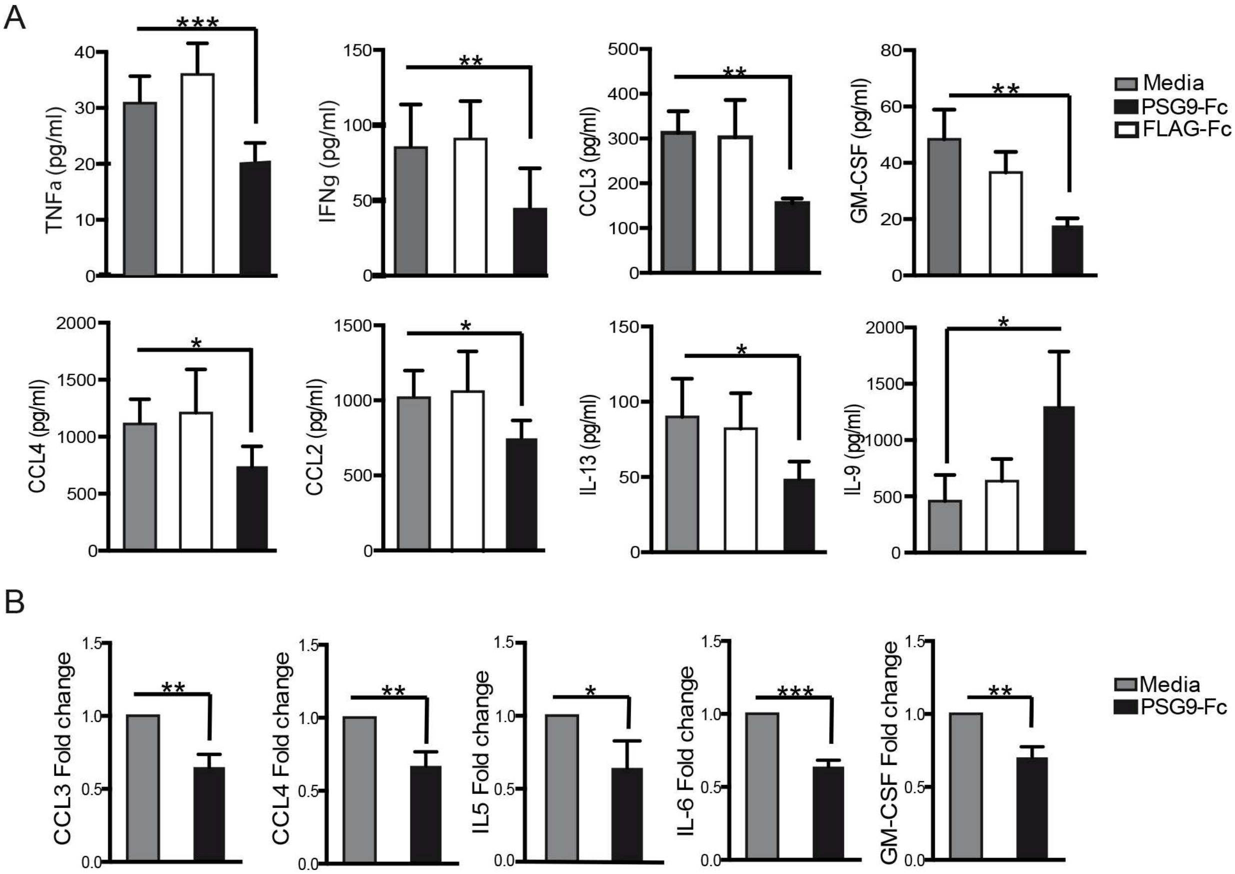

Fig 8. Pro-inflammatory cytokine expression is reduced upon T-cell treatment with PSG9. Supernatants from naive CD4+ mouse (A) or human (B) T-cells were collected 72 h after activation and treatment with PSG9 or FLAG. Cytokine concentrations were measured using multiplex immunoassay kits with the Bioplex system. P values were calculated by Student's *t*-tests. *P<0.05, **P<0.005, ***P<0.0005, ****P<0.0001.

doi:10.1371/journal.pone.0158050.g008

the PSG9-LAP interaction estimated in 0.5 μM. The dissociation constant for PSG9 with LAP was also found to be small (kd = 0.53 μM), resulting in prolonged half-life time for the PSG9-LAP complex. Further, we found that PSG9 does not bind to LAP via the same amino acid sequence as TSP-1, indicating that PSG9 activates TGF-β1 through a different mechanism than TSP-1.

The results presented in Fig 3 show that PSG9 activates the SLC of TGF-β1 in a dose-dependent manner using two different strategies, one based on an ELISA that only detects the active form of the cytokine and the well-characterized luciferase reporter cell line (MLEC) that allows quantification of active TGF-β1. Therefore PSG9 can activate the latent complex that is not anchored to the extracellular matrix, as required by other activators of this cytokine, and could also potentially interact with the large latent complex (LLC) of TGF-β1 which constitutes an anchored reservoir of the latent form of this cytokine [51,52].

The N-domain of PSG1 induces the secretion of latent TGF-β1 from macrophages and the amino acids $L_{41}Y_{42}H_{43}Y_{44}$ in the CC' loop in this domain are required for this activity [23]. These amino acids are conserved in the N-domain of PSG9 and therefore it was not surprising to find that treatment of the macrophage cell line RAW264.7 with a recombinant protein consisting of the N-domain of PSG9 (PSG9N-Fc) had the same activity. Pharmacological inhibition of the TGF-β receptor ALK 5 did not inhibit the PSG9N-mediated increase in TGF-β

secretion by RAW264.7 cells. Therefore autostimulation by TGF-β is not involved in the observed response. On the other hand, the possibility exists that, following activation of latent TGF-β by full length PSG9, the active form of the cytokine could be involved in a positive feedback loop resulting in even higher secretion of TGF-β by macrophages than what is observed upon treatment with the N-domain of the protein. We previously reported that the B2-domain of another human PSG family member, PSG1, binds to LAP of TGF-β1 with much higher affinity when compared to the other domains of the protein [23]. While we have not determined whether the B2 domain of PSG9 is the major domain responsible for the ability of PSG9 to activate TGF-β1, we observed that while the protein composed of only the N-domain was able to activate TGF-β1 as measured by the TGF-β luciferase reporter cell line, it did so at much higher concentrations than the full length protein. This strongly suggests that at least one other domain of the protein is implicated in this PSG9 function. We expanded these studies to determine whether PSG9N-Fc could induce the secretion of TGF-β1 from activated mouse and human $CD4^+$ T-cells. As expected, we found that activated $CD4^+$ T-cells secrete latent TGF-β1 but the amount of TGF-β1 produced by the cells was not further increased by the addition of PSG9N-Fc. At this time, since the receptor for the N-domain of PSG9 that mediates the action of this protein in macrophages remains to be identified, we cannot determine whether the potential absence of the receptor or difference in the signaling events following its engagement are responsible for the inability of this protein to induce the secretion of TGF-β1 by $CD4^+$ T-cells.

TGF-β1 is essential for the differentiation of $FoxP3^+$ regulatory T-cells, which are crucial for maintenance of immune tolerance and prevention of autoimmune reactions [53]. Although PSG9 is not able to induce additional secretion of TGF-β1 by $CD4^+$ T-cells, we observed that PSG9 is able to activate latent TGF-β1. Consequently we hypothesized that PSG9 might be sufficient to promote the conversion of naïve $CD4^+$ T-cells into Foxp3-expressing regulatory T-cells. Naïve $CD4^+$ T-cells isolated from the spleen of Foxp3-IRES-GFP knock-in mice and $CD4^+CD25^-$T-cells isolated from peripheral blood of healthy volunteers were employed to test this hypothesis. We found that treatment of cells with PSG9 increased the percentage of $CD4^+FoxP3^+$ expressing cells and that this increase was dose and TGF-β1-dependent as it did not occur in the presence of the TGF-β receptor I inhibitor SB431542. In addition, we found that PSG9 increased the percentage of human $CD4^+$ T-cells expressing cell surface LAP. $CD4^+$ Foxp3 $^-LAP^+$ regulatory T-cells are highly dependent on TGF-β1 and have been shown to block allergic inflammation of the lungs [20,54].

TGF-β suppresses the transcription of IL-2 in T-cells induced by TCR signals, which constitutes an important process of immune regulation mediated by this cytokine [37]. We found that murine activated $CD4^+$ T-cells treated with PSG9 secreted lower amount of IL-2 than protein control- or media only- treated cells. Once more, this PSG9 activity was dependent on the generation of active TGF-β1 and was dose-dependent. Furthermore, we observed that suppression of IL-2 secretion was not the result of consumption of the cytokine by the cells but the result of inhibition of transcription. We then explored whether PSG9 treatment could regulate the secretion of other pro-inflammatory chemokines and cytokines by murine and human activated T-cells. We found that several pro-inflammatory cytokines, including TNF-α, IFNγ and the chemokines CCL2 and CCL-4, were reduced in cells treated with PSG9. In addition, IL-9, an important anti-inflammatory cytokine, was increased in mouse cells treated with PSG9. Overall, PSG9 is able to prevent increases in pro-inflammatory cytokine release in human and mouse cells and promoted the levels of tolerance-inducing IL-9 in mice.

Our results show that PGS9 activates TGF-β1 and increases the number and percentage of $CD4^+$ T-cells with regulatory function, further supporting the role of PSG9 in immune

tolerance. These observations suggest that PSG9 should be considered as a novel candidate for the treatment of autoimmune diseases and for the establishment of immune tolerance.

Supporting Information

S1 File. Data sets for Figs 3–8.
(XLSX)

S2 File. Data set for Fig 2.
(PPTX)

S3 File. Data set for supporting information. RAW264.7 cells were treated with PSG9N in the presence of SB-431542, and showed a significant increase in TGF-β1 in the cell supernatant when compared to protein-control treated cells, indicating that the observed increase in TGF-β1 is not due to a positive feedback loop. Additional supporting information for FACS data for Figs 5 and 6 is available at https://figshare.com/s/dea1406c002c1d6ea7d8.
(XLSX)

Acknowledgments

We would like to thank Dr. Wolfgang Zimmermann for helpful discussions.

Author Contributions

Conceived and designed the experiments: KJ AB HM EK GD. Performed the experiments: KJ AB JW SR MM. Analyzed the data: KJ AB EK GD. Contributed reagents/materials/analysis tools: EK GD. Wrote the paper: KJ AB HM GD.

References

1. Jurisicova A, Antenos M, Kapasi K, Meriano J, Casper RF (1999) Variability in the expression of trophectodermal markers beta-human chorionic gonadotrophin, human leukocyte antigen-G and pregnancy specific beta-1 glycoprotein by the human blastocyst. Hum Reprod 14: 1852–1858. PMID: 10402404
2. Horne CH, Towler CM, Pugh-Humphreys RG, Thomson AW, Bohn H (1976) Pregnancy specific beta1-glycoprotein—a product of the syncytiotrophoblast. Experientia 32: 1197. PMID: 971765
3. Towler CM, Horne CH, Jandial V, Campbell DM, MacGillivray I (1976) Plasma levels of pregnancy-specific beta1-glycoprotein in normal pregnancy. Br J Obstet Gynaecol 83: 775–779. PMID: 990216
4. Martinez FF, Cervi L, Knubel CP, Panzetta-Dutari GM, Motran CC (2013) The role of pregnancy-specific glycoprotein 1a (PSG1a) in regulating the innate and adaptive immune response. Am J Reprod Immunol 69: 383–394. doi: 10.1111/aji.12089 PMID: 23432664
5. Falcon CR, Martinez FF, Carranza F, Cervi L, Motran CC (2014) In Vivo Expression of Recombinant Pregnancy-Specific Glycoprotein 1a Inhibits the Symptoms of Collagen-Induced Arthritis. Am J Reprod Immunol 72: 527–533. doi: 10.1111/aji.12307 PMID: 25112392
6. Blois SM, Sulkowski G, Tirado-Gonzalez I, Warren J, Freitag N, Klapp BF, et al. (2014) Pregnancy-specific glycoprotein 1 (PSG1) activates TGF-beta and prevents dextran sodium sulfate (DSS)-induced colitis in mice. Mucosal Immunol 7: 348–358. doi: 10.1038/mi.2013.53 PMID: 23945545
7. Salem HT, Lee JN, Seppala M, Vaara L, Aula P, Al-Ani AT, et al. (1981) Measurement of placental protein 5, placental lactogen and pregnancy-specific beta 1 glycoprotein in mid-trimester as a predictor of outcome of pregnancy. Br J Obstet Gynaecol 88: 371–374. PMID: 6971651
8. Wurz H, Geiger W, Kunzig HJ, Jabs-Lehmann A, Bohn H, Luben G (1981) Radioimmunoassay of SP1 (pregnancy-specific beta1-glycoprotein) in maternal blood and in amniotic fluid normal and pathologic pregnancies. J Perinat Med 9: 67–78. PMID: 6787188
9. Pihl K, Larsen T, Laursen I, Krebs L, Christiansen M (2009) First trimester maternal serum pregnancy-specific beta-1-glycoprotein (SP1) as a marker of adverse pregnancy outcome. Prenat Diagn 29: 1256–1261. doi: 10.1002/pd.2408 PMID: 19911417

10. Olsen A, Teglund S, Nelson D, Gordon L, Copeland A, Georgescu A, et al. (1994) Gene organization of the pregnancy-specific glycoprotein region on human chromosome 19: assembly and analysis of a 700-kb cosmid contig spanning the region. Genomics 23: 659–668. PMID: 7851895

11. Chang CL, Semyonov J, Cheng PJ, Huang SY, Park JI, Tsai H-J, et al. (2013) Widespread Divergence of the CEACAM/PSG Genes in Vertebrates and Humans Suggests Sensitivity to Selection. PLoS One 8: e61701. doi: 10.1371/journal.pone.0061701 PMID: 23613906

12. Sudmant PH, Rausch T, Gardner EJ, Handsaker RE, Abyzov A, Huddleston J, et al. (2015) An integrated map of structural variation in 2,504 human genomes. Nature 526: 75–81. doi: 10.1038/nature15394 PMID: 26432246

13. Dumont BL, Eichler EE (2013) Signals of historical interlocus gene conversion in human segmental duplications. PLoS One 8: e75949. doi: 10.1371/journal.pone.0075949 PMID: 24124524

14. Teglund S, Zhou GQ, Hammarstrom S (1995) Characterization of cDNA encoding novel pregnancy-specific glycoprotein variants. Biochem Biophys Res Commun 211: 656–664. PMID: 7794280

15. Camolotto S, Racca A, Rena V, Nores R, Patrito LC, Genti-Raimondi S, et al. (2010) Expression and transcriptional regulation of individual pregnancy-specific glycoprotein genes in differentiating trophoblast cells. Placenta 31: 312–319. doi: 10.1016/j.placenta.2010.01.004 PMID: 20116096

16. Shanley DK, Kiely PA, Golla K, Allen S, Martin K, O'Riordan RT, et al. (2013) Pregnancy-specific glycoproteins bind integrin alphaIIbbeta3 and inhibit the platelet-fibrinogen interaction. PLoS One 8: e57491. doi: 10.1371/journal.pone.0057491 PMID: 23469002

17. Blankley RT, Fisher C, Westwood M, North R, Baker PN, Walker MJ, et al. (2013) A label-free SRM workflow identifies a subset of pregnancy specific glycoproteins as potential predictive markers of early-onset pre-eclampsia. Mol Cell Proteomics.

18. Blois SM, Sulkowski G, Tirado-Gonzalez I, Warren J, Freitag N, Klapp BF, et al. (2014) Pregnancy-specific glycoprotein 1 (PSG1) activates TGF-beta and prevents dextran sodium sulfate (DSS)-induced colitis in mice. Mucosal Immunol 7: 3448–3458.

19. Ballesteros A, Mentink-Kane MM, Warren J, Kaplan GG, Dveksler GS (2015) Induction and activation of latent transforming growth factor-beta1 are carried out by two distinct domains of pregnancy-specific glycoprotein 1 (PSG1). J Biol Chem 290: 4422–4431. doi: 10.1074/jbc.M114.597518 PMID: 25548275

20. Gandhi R, Farez MF, Wang Y, Kozoriz D, Quintana FJ, Weiner HL (2010) Cutting edge: human latency-associated peptide+ T cells: a novel regulatory T cell subset. J Immunol 184: 4620–4624. doi: 10.4049/jimmunol.0903329 PMID: 20368276

21. Lisboa FA, Warren J, Sulkowski G, Aparicio M, David G, Zudaire E, et al. (2011) Pregnancy-specific glycoprotein 1 induces endothelial tubulogenesis through interaction with cell surface proteoglycans. J Biol Chem 286: 7577–7586. doi: 10.1074/jbc.M110.161810 PMID: 21193412

22. Abe M, Harpel JG, Metz CN, Nunes I, Loskutoff DJ, Rifkin DB (1994) An assay for transforming growth factor-beta using cells transfected with a plasminogen activator inhibitor-1 promoter-luciferase construct. Anal Biochem 216: 276–284. PMID: 8179182

23. Ballesteros A, Mentink-Kane MM, Warren J, Kaplan GG, Dveksler GS (2015) Induction and activation of latent TGF-beta1 is carried out by two distinct domains of Pregnancy-Specific Glycoprotein 1. J Biol Chem 290: 4422–4431. doi: 10.1074/jbc.M114.597518 PMID: 25548275

24. Wakefield LM, Smith DM, Broz S, Jackson M, Levinson AD, Sporn MB (1989) Recombinant TGF-beta 1 is synthesized as a two-component latent complex that shares some structural features with the native platelet latent TGF-beta 1 complex. Growth Factors 1: 203–218. PMID: 2629880

25. Annes JP, Munger JS, Rifkin DB (2003) Making sense of latent TGFbeta activation. J Cell Sci 116: 217–224. PMID: 12482908

26. Sweetwyne MT, Murphy-Ullrich JE (2012) Thrombospondin1 in tissue repair and fibrosis: TGF-beta-dependent and independent mechanisms. Matrix Biol 31: 178–186. doi: 10.1016/j.matbio.2012.01.006 PMID: 22266026

27. Young GD, Murphy-Ullrich JE (2004) Molecular interactions that confer latency to transforming growth factor-beta. J Biol Chem 279: 38032–38039. PMID: 15208302

28. Murphy-Ullrich JE, Poczatek M (2000) Activation of latent TGF-beta by thrombospondin-1: mechanisms and physiology. Cytokine Growth Factor Rev 11: 59–69. PMID: 10708953

29. Snyder SK, Wessner DH, Wessells JL, Waterhouse RM, Wahl LM, Zimmermann W, et al. (2001) Pregnancy-specific glycoproteins function as immunomodulators by inducing secretion of IL-10, IL-6 and TGF-beta1 by human monocytes. Am J Reprod Immunol 45: 205–216. PMID: 11327547

30. Chen W, Jin W, Hardegen N, Lei KJ, Li L, Marinos N, et al. (2003) Conversion of peripheral CD4+CD25-naive T cells to CD4+CD25+ regulatory T cells by TGF-beta induction of transcription factor Foxp3. J Exp Med 198: 1875–1886. PMID: 14676299

31. Lu L, Wang J, Zhang F, Chai Y, Brand D, Wang X, et al. (2010) Role of SMAD and non-SMAD signals in the development of Th17 and regulatory T cells. J Immunol 184: 4295–4306. doi: 10.4049/jimmunol.0903418 PMID: 20304828

32. Tone Y, Furuuchi K, Kojima Y, Tykocinski ML, Greene MI, Tone M (2008) Smad3 and NFAT cooperate to induce Foxp3 expression through its enhancer. Nat Immunol 9: 194–202. PMID: 18157133

33. Marie JC, Letterio JJ, Gavin M, Rudensky AY (2005) TGF-beta1 maintains suppressor function and Foxp3 expression in CD4+CD25+ regulatory T cells. J Exp Med 201: 1061–1067. PMID: 15809351

34. Xu L, Kitani A, Fuss I, Strober W (2007) Cutting edge: regulatory T cells induce CD4+CD25-Foxp3- T cells or are self-induced to become Th17 cells in the absence of exogenous TGF-beta. J Immunol 178: 6725–6729. PMID: 17513718

35. Tran DQ, Andersson J, Wang R, Ramsey H, Unutmaz D, Shevach EM (2009) GARP (LRRC32) is essential for the surface expression of latent TGF-beta on platelets and activated FOXP3+ regulatory T cells. Proc Natl Acad Sci U S A 106: 13445–13450. doi: 10.1073/pnas.0901944106 PMID: 19651619

36. Stockis J, Colau D, Coulie PG, Lucas S (2009) Membrane protein GARP is a receptor for latent TGF-beta on the surface of activated human Treg. Eur J Immunol 39: 3315–3322. doi: 10.1002/eji.200939684 PMID: 19750484

37. Wakabayashi Y, Tamiya T, Takada I, Fukaya T, Sugiyama Y, Inoue N, et al. (2011) Histone 3 lysine 9 (H3K9) methyltransferase recruitment to the interleukin-2 (IL-2) promoter is a mechanism of suppression of IL-2 transcription by the transforming growth factor-beta-Smad pathway. J Biol Chem 286: 35456–35465. doi: 10.1074/jbc.M111.236794 PMID: 21862595

38. Bettelli E, Carrier Y, Gao W, Korn T, Strom TB, Oukka M, et al. (2006) Reciprocal developmental pathways for the generation of pathogenic effector TH17 and regulatory T cells. Nature 441: 235–238. PMID: 16648838

39. Veldhoen M, Hocking RJ, Atkins CJ, Locksley RM, Stockinger B (2006) TGFbeta in the context of an inflammatory cytokine milieu supports de novo differentiation of IL-17-producing T cells. Immunity 24: 179–189. PMID: 16473830

40. Coghill JM, Sarantopoulos S, Moran TP, Murphy WJ, Blazar BR, Serody JS (2011) Effector CD4+ T cells, the cytokines they generate, and GVHD: something old and something new. Blood 117: 3268–3276. doi: 10.1182/blood-2010-12-290403 PMID: 21245483

41. Han KL, Thomas SV, Koontz SM, Changpriroa CM, Ha SK, Malech HL, et al. (2013) Adenosine A(2)A receptor agonist-mediated increase in donor-derived regulatory T cells suppresses development of graft-versus-host disease. J Immunol 190: 458–468. doi: 10.4049/jimmunol.1201325 PMID: 23225892

42. Chatterjee P, Chiasson VL, Bounds KR, Mitchell BM (2014) Regulation of the Anti-Inflammatory Cytokines Interleukin-4 and Interleukin-10 during Pregnancy. Front Immunol 5: 253. doi: 10.3389/fimmu.2014.00253 PMID: 24904596

43. Lu LF, Lind EF, Gondek DC, Bennett KA, Gleeson MW, Pino-Lagos K, et al. (2006) Mast cells are essential intermediaries in regulatory T-cell tolerance. Nature 442: 997–1002. PMID: 16921386

44. Kulkarni AB, Huh CG, Becker D, Geiser A, Lyght M, Flanders KC, et al. (1993) Transforming growth factor beta 1 null mutation in mice causes excessive inflammatory response and early death. Proc Natl Acad Sci U S A 90: 770–774. PMID: 8421714

45. Bennett CL, Christie J, Ramsdell F, Brunkow ME, Ferguson PJ, Whitesell L, et al. (2001) The immune dysregulation, polyendocrinopathy, enteropathy, X-linked syndrome (IPEX) is caused by mutations of FOXP3. Nat Genet 27: 20–21. PMID: 11137993

46. Fontenot JD, Rasmussen JP, Williams LM, Dooley JL, Farr AG, Rudensky AY (2005) Regulatory T cell lineage specification by the forkhead transcription factor foxp3. Immunity 22: 329–341. PMID: 15780990

47. Chen Z, Herman AE, Matos M, Mathis D, Benoist C (2005) Where CD4+CD25+ T reg cells impinge on autoimmune diabetes. J Exp Med 202: 1387–1397. PMID: 16301745

48. Aluvihare VR, Kallikourdis M, Betz AG (2004) Regulatory T cells mediate maternal tolerance to the fetus. Nat Immunol 5: 266–271. PMID: 14758358

49. Bailly S, Brand C, Chambaz EM, Feige JJ (1997) Analysis of small latent transforming growth factor-beta complex formation and dissociation by surface plasmon resonance. Absence of direct interaction with thrombospondins. J Biol Chem 272: 16329–16334. PMID: 9195938

50. Radaev S, Zou Z, Huang T, Lafer EM, Hinck AP, Sun PD (2010) Ternary complex of transforming growth factor-beta1 reveals isoform-specific ligand recognition and receptor recruitment in the superfamily. J Biol Chem 285: 14806–14814. doi: 10.1074/jbc.M109.079921 PMID: 20207738

51. Annes JP, Chen Y, Munger JS, Rifkin DB (2004) Integrin alphaVbeta6-mediated activation of latent TGF-beta requires the latent TGF-beta binding protein-1. J Cell Biol 165: 723–734. PMID: 15184403

52. Todorovic V, Jurukovski V, Chen Y, Fontana L, Dabovic B, Rifkin DB (2005) Latent TGF-beta binding proteins. Int J Biochem Cell Biol 37: 38–41. PMID: 15381147

53. Tran DQ (2012) TGF-beta: the sword, the wand, and the shield of FOXP3(+) regulatory T cells. J Mol Cell Biol 4: 29–37. doi: 10.1093/jmcb/mjr033 PMID: 22158907

54. Duan W, So T, Mehta AK, Choi H, Croft M (2011) Inducible CD4+LAP+Foxp3- regulatory T cells suppress allergic inflammation. J Immunol 187: 6499–6507. doi: 10.4049/jimmunol.1101398 PMID: 22079987
